# Quantitative Morphometric, Physiological, and Metabolic Characteristics of Chickens and Mallards for Physiologically Based Kinetic Model Development

**DOI:** 10.3389/fphys.2022.858283

**Published:** 2022-04-06

**Authors:** Colin G. Scanes, Johannes Witt, Markus Ebeling, Stephan Schaller, Vanessa Baier, Audrey J. Bone, Thomas G. Preuss, David Heckmann

**Affiliations:** ^1^Department of Poultry Science, University of Arkansas, Fayetteville, AR, United States; ^2^Department of Biological Science, University of Wisconsin Milwaukee, Milwaukee, WI, United States; ^3^Bayer Crop Science, Monheim am Rhein, Germany; ^4^esqLABS, GmbH, Saterland, Germany; ^5^Bayer Crop Science, Chesterfield, MO, United States

**Keywords:** quantative, morphometric, chickens, mallards, physiological, metabolic

## Abstract

Physiologically based kinetic (PBK) models are a promising tool for xenobiotic environmental risk assessment that could reduce animal testing by predicting *in vivo* exposure. PBK models for birds could further our understanding of species-specific sensitivities to xenobiotics, but would require species-specific parameterization. To this end, we summarize multiple major morphometric and physiological characteristics in chickens, particularly laying hens (*Gallus gallus*) and mallards (*Anas platyrhynchos*) in a meta-analysis of published data. Where such data did not exist, data are substituted from domesticated ducks (*Anas platyrhynchos*) and, in their absence, from chickens. The distribution of water between intracellular, extracellular, and plasma is similar in laying hens and mallards. Similarly, the lengths of the components of the small intestine (duodenum, jejunum, and ileum) are similar in chickens and mallards. Moreover, not only are the gastrointestinal absorptive areas similar in mallard and chickens but also they are similar to those in mammals when expressed on a log basis and compared to log body weight. In contrast, the following are much lower in laying hens than mallards: cardiac output (CO), hematocrit (Hct), and blood hemoglobin. There are shifts in ovary weight (increased), oviduct weight (increased), and plasma/serum concentrations of vitellogenin and triglyceride between laying hens and sexually immature females. In contrast, reproductive state does not affect the relative weights of the liver, kidneys, spleen, and gizzard.

## Introduction

One of the principal avian species employed generating data for pesticide registration is mallards (*Anas platyrhynchos*; U.S. Environmental Protection Agency, 2004; de Montaigu and Goulson, 2020). Laying hens have been employed to assess safety of veterinary drugs and pesticides (Goetting et al., 2011). With the moves to reduce use of test avian species for pesticide registration (e.g., U.S. Environmental Protection Agency, 2019), there is a need to have alternative computational models. Physiologically based kinetic (PBK) models are one example for such tools. By incorporating a mechanistic description of physiological compartments, PBK models can predict the absorption, distribution, metabolism, and excretion (ADME) of xenobiotics. Within PBK models, physiology of the species and compound-specific properties of the ADME process are treated separately. Therefore, PBK models require a species-specific parameterization of physiology. Examples of application in risk assessment include dose extrapolation, exposure scenario extrapolation, or interspecies extrapolation (see, e.g., OECD, 2021). PBK models have been developed for the chicken (Lautz et al., 2020), and compound-specific models have been published for the American kestrel (Nichols et al., 2010), turkey, and quail (Cortright et al., 2009). Despite these advances, many ecologically important species are still lacking and parameterization of such models remains challenging, especially in the case of many endangered and focal species. Thus, there is the need for widely available morphological and physiological parameters to support the development of species-specific PBK models.

The present communication outlines quantitative anatomical and physiological parameters in chickens (particularly laying hens) and mallards based on a meta-analysis of the literature. There is a recent paper providing a meta-analysis of series of physiological and anatomical parameters in laying hens (Wang et al., 2020) but neither mallards nor domestic ducks (both *Anas platyrhynchos*). It is argued that the present report is broadly complementary but based a much greater number of reports providing greater confidence in the accuracy for each parameter. It is noted that there are also several parameters where employing greater numbers of studies shifts the numerical value of parameter markedly. Moreover, there are parameters in the present study that were not included in the Wang et al. (2020) analysis.

The present communication outlines quantitative anatomical and physiological parameters in chickens (particularly laying hens) and mallards based on a meta-analysis of the literature. There was limited information on physiological and morphometric parameters in mallard ducks. In these cases, and where data are available for sexually immature female chickens, parameterization data from laying hens were compared with those in sexually immature female chickens. Similarities in the data from laying hens and sexually immature female chickens would be indicative of the parameterization data being robust and is suggestive that the data from chicken may be applied to mallard ducks. In contrast, the data or laying hens need to be treated with some caution where there are differences between the data from laying hen and sexually immature female chickens; the caveat being that there are shifting in physiology accompanying egg laying. For instance, the liver will be producing yolk precursors. In addition, the oviduct will undergo considerable growth and maturation to synthesize and deposit egg albumen around the yolk filled oocyte.

In cases where there were either a lack of published data on sexually immature chickens or marked differences in the data between different studies (in Supplementary Material) and consequently a high coefficient of variation, the robustness of the data was further tested by comparison with young broiler chickens. It is noted that there are genetic differences between lines of chickens that are used to produce eggs and those used for chicken meat. Similarities between parameterization data from laying hens and young broiler chickens would also indicate robustness of the data from laying hens. Similarly, the validity of the parameterization data for mallard ducks, where available, was tested by comparison of mallard data with data in domesticated ducks. This also provided evidence for or against domestication *per se* influencing the parameters. This is important as the laying hen is used as a surrogate for wild birds.

There is a recent paper providing a meta-analysis of series of physiological and anatomical parameters in laying hens (Wang et al., 2020) but neither mallards nor domestic ducks (both *Anas platyrhynchos*). It is argued that the present report is broadly complementary but it based a much greater number of reports. This provides greater confidence in the accuracy for each parameter. It is noted that there are also several parameters where employing greater numbers of studies shifts the numerical value of parameter markedly. Moreover, there are parameters in the present study that were not included in the analysis of Wang et al. (2020).

## Materials and Methods

### Development of Data Bases for Physiological, Biochemical, and Morphological Characteristics

Data bases were developed based on published reports. Among the search terms employed were the following:

- Hematocrit (Hct) AND laying hens (or pullets or chickens or broilers or mallards or ducks) AND scholarly
- Hemoglobin AND laying hens (or pullets or chickens or broilers or mallards or ducks) AND scholarly
- Plasma AND protein (or albumin or immunoglobulin or lipid or triglyceride or phospholipid or cholesterol) AND laying hens (or pullets or mallards or ducks) AND scholarly
- Ovary (or uterus/oviduct or lungs or kidneys or heart or other organs) AND laying hens (or pullets) AND scholarly
- Small intestine AND laying hens (or pullets or chickens or mallards or ducks) AND scholarly
- Villus AND laying hens (or pullets or chickens or mallards or ducks) AND scholarly
- Cardiac output (CO) AND laying hens (or pullets or chickens or mallards or ducks) AND scholarly
- Liver cellularity (or lipids) AND laying hens (or pullets or chickens or mallards or ducks) AND scholarly

This was supplemented by cross checking the cited papers in the top 15 papers identified by the initial search.

Cell volume (liver and small intestine mucosa) and cell number were calculated from the published hepatic DNA concentrations and the mass of DNA in female chickens (Mendonça et al., 2010, 2016). The number of liver cells per gram was calculated as follows: DNA concentration (mg per gram)/diploid genome mass in mg. This was simplified as DNA concentration as mg per gram × 10^9^/diploid genome mass in pg. We employed a mass of 2.15 pg for chicken diploid genome (Mendonça et al., 2010, 2016). The mass of cells was calculated as 1/number of cells per gram in pg. The volume was assumed to be numerically equal to the mass × 10^−3^. The calculations assumed that the tissue has a density of 1.0 kg L^−1^.

### Bird Ages

Sexually mature female birds were those that were laying eggs either in domesticated species or during the breeding season for wild birds. Data for sexually immature birds included in the analysis were restricted to birds that were over 75% of mature body weight and were neither laying eggs nor incubating eggs.

### Gastrointestinal Absorptive Area

Estimation of the gastro-intestinal absorptive area entailed the following:

1. Estimation of the nominal surface area of intestinal components assuming them to be cylindrical (Area = π. 2. Radius. Length).
2. Correction for the presence of villi in the form of the villus amplification factor (VAF) with the VAF = 1 + [π × L′ × 2R′× D′] Where L′ is villus length (μm), 2R′ [2 × radius of villus or villus diameter (μm) and D′ is villus density (number per μm^2^); Ferraris et al., 1989; Ferrer et al., 1995]. (Note: although MAF is dimensionless, the dimensions of the length, radius, and density must be the same units, specifically μm and μm^2^.) This assumes that villi are circular cylinders.
3. Correction for the presence of microvilli in the form of the microvillus amplification factor (MAF) with MAF = 1 + [π × L × 2R × D] where L is microvillus length (μm), 2R (2 × radius) is microvillus diameter (μm), and D is microvillus density (number per μm^2^ or per 10^−12^ m^2^; Ferraris et al., 1989; Ferrer et al., 1995). [Note: although MAF is dimensionless, the dimensions of the length, radius, and density must be the same units, specifically μm and μm^2^.] This also assumes that microvilli are circular cylinders.

### Statistical Analysis

Data (the mean from the individual studies) were analyzed by paired and unpaired Students *t* tests or by one-way ANOVA followed by Dunnett’s range test employing Excel software (Microsoft Excel 2019 for Mac).

## Results

### Fluid Dynamics in Adult Female Chickens or Ducks

Table 1 summarizes fluid dynamics in laying hens (*Gallus gallus*) and mallards (*Anas platyrhynchos*; or domesticated ducks—*Anas platyrhynchos*). Extracellular water, plasma, and interstitial fluid, expressed as a percent of body weight are similar in laying hens and mallards (Table 1). There appear to be differences in laying hens and mallards for intracellular water and total water expressed as a percentage of body weight.

**Table 1:** Fluid dynamics in adult female chickens or mallards.

Parameter	Mean ± (*n* = studies) SEM	
	Laying hen (*Gallus gallus*)	Mallards (*Anas platyrhynchos*)
Water		
Total body water % of b.wt.	57.3^A^	68.5^C^
Intracellular water % of b.wt.	31.1^A^	44.8^C^
Extracellular water % of b.wt.	26.2^A^	23.7^C^
Plasma % of body weight	4.6^A^	5.3^B^
Interstitial fluid % of b.wt.	21.7^A^	17.9^C^
Heart		
Cardiac Output (CO) ml min^−1^	316 ± (7) 35.6^D^	1,203 (2)^D^
CO ml min^−1^ kg^−1^ (b.wt.)	176 ± (7) 16.0^D^^E^	416 (2)^D^
Weight % of b.wt.	0.446 ± (9) 0.040^F^	1.13^G^
Circulatory system		
Hematocrit %	27.1 ± (10) 0.86^H^	45.4 ± (4) 1.82^H^^**^
Blood hemoglobin g dL^−1^	9.2 ± (5) 0.62^H^	15.4 ± (3) 0.93^H^^**^
Hepatic portal blood flow ml min^−1^	32.8^I^	NA
Hepatic portal blood flow ml kg^−1^ min^−1^	14.8^I^	NA

A *Medway and Kare (1959)*.

B *Mean of Keijer and Butler (1982) and Roberts and Hughes (1984)*.

C *Roberts and Hughes (1984)*.

D *Means of individual studies in Supplementary Table 1 of Supplementary Data*.

E *Estimated based on the assumption that laying hens weigh 1.6 kg*.

F *Means of individual studies in Supplementary Table 7 of Supplementary Data*.

G *Krapo (1981)*.

H *Means of individual studies in Supplementary Table 4 of Supplementary Data*.

I *Based on studies employing non-anaesthetized fed adult males (Sturkie and Abati, 1975)*.

*NA, not available*.

** *Difference between species *p* < 0.01*.

The CO (ml kg^−1^ min^−1^) in laying hens (Table 1) is also very similar to that in young (broiler) chickens 192 ± (5) 5.6 ml kg^−1^ min^−1^ (for means of individual studies see Supplementary Table 1 in Supplementary Data). The CO is markedly greater in mallards than laying hens (Table 1).

### Blood

#### Hematocrit/Packed Cell Volume

The Hct/packed cell volume (PCV) is 40.3% lower (*p* < 0.0001) in adult female chickens (laying hens) than in mallard ducks (Table 1). To provide support for the veracity of the Hct/PCV, the Hct/PCV was compared between mallard ducks and domesticated ducks. Hct/PCV did not differ (*p* > 0.15) between mallard ducks (Table 1) and domesticated ducks [41.0 ± (9) 1.30%]. Similarly, there was no difference between Hct/PCV in sexually immature pullets and laying hens (see Table 2; see Supplementary Table 2 for data on individual studies). The Hct/PCV was also low in young broiler chickens [4–6 -week-old male and females; 31.4 ± (9) 3.24%] albeit slightly higher (*p* < 0.05) than in laying hens (see Supplementary Table 2 for data on individual studies). The Hct/PCV was 32.1% lower (*p* < 0.003) in laying hens than adult male chickens [39.9 ± (3) 3.24%; see Supplementary Table 2 for data on individual studies].

**Table 2:** Comparison of characteristics of blood in sexually immature females (pullets) and laying chickens (*Gallus gallus*).

Parameter	Mean ± (*n* = studies) SEM	
	Sexually immature females (pullets)	Mature laying hens
Hematocrit/PCV %	28.1 ± (4) 1.78^A^	27.1 ± (10) 0.86^A^
Hemoglobin %	8.88 ± (2) 0.38^A^	9.18 ± (5) 0.62^A^
Plasma protein g dL^−1^	3.80 ± (2) 1.0^B^	4.53 ± (3) 0.52^B^
Albumin g dL^−1^	1.67 ± (5) 0.098^C^	1.19 ± (5) 0.18^C^^*^
IgY g dL^−1^	0.948 (1)^C^	3.66 ± (3) 1.47^C^
IgA g dL^−1^	0.342^C^	0.323^C^
IgM g dL^−1^	NA	1.32 ± (2) 0.40^C^
Vitellogenin g dL^−1^	0^D^	1.63^D^
Triglyceride g dL^−1^	0.408 ± (3) 0.063^E^	2.268 ± (5) 0.516^E^^*^
Cholesterol g dL^−1^	0.101 ± (3) 0.0023^E^	0.172 ± (5) 0.064^E^
Phospholipids g dL^−1^	0.141 ± (3) 0.040^F^	0.453 ± (3) 0.247^F^

* *Difference with sexually immature female chickens *p* < 0.05*.

A *For data from different studies see Supplementary Tables 2, 3*.

B *Sturkie and Newman (1951); Morgan (1975); Yuan et al. (2016); and Rezende et al. (2017)*.

C *For data from different studies, See Supplementary Table 5*.

D *Redshaw and Follett (1976)*.

E *Neill et al. (1977); Hagan et al. (1984); Lien et al. (2001); Peebles et al. (2004); and Lv et al. (2018)*.

F *Neill et al. (1977) and Hagan et al. (1984)*.

*NA, not available*.

#### Hemoglobin

Blood hemoglobin concentrations were 40.2% lower (*p* < 0.002) in laying hens than in mallards (Table 1). There were no differences in the hemoglobin concentrations between sexually immature and mature female chickens (Table 2) or between laying hens and young broiler chickens [9.33 ± (6) 0.63; Table 2; see Supplementary Tables 2, 3 for data on individual studies]. Similarly, there was no difference between the blood concentration of hemoglobin in mallards (Table 1) and those in domesticated ducks [13.3 ± (6) 0.30 g dL^−1^; see Supplementary Table 3 for data on individual studies].

#### Plasma/Serum Concentrations of Total Protein

There were similar serum/plasma total protein concentrations in non-reproductive female and male mallards [respectively, 4.40 ± (55 birds) 0.10 g dL^−1^; 4.11 ± (26) 0.17 g dL^−1^ calculated from Driver, 1981; Fairbrother et al., 1990]. These were also consistent with those in sexually immature female chickens (Table 2). Serum/plasma protein total concentrations were increased 43.2% (*p* < 0.001) between non-reproductive female mallards and laying female mallards [to 6.30 ± (18 birds) 0.28 g dL^−1^ calculated from Fairbrother et al., 1990].

The increase in total protein in the serum/plasma in mallards between reproductively quiescent and laying females (Δ = 1.9 g dL^−1^) is quantitatively similar to the increase in circulating concentration of vitellogenin (Table 2).

#### Plasma/Serum Concentrations of Albumin

Serum/plasma concentrations of albumin were 28.7% lower (*p* = 0.0168) in laying hens than in young chickens (Table 2; also see Supplementary Table 5). Two reports are excluded from the analysis of plasma/serum albumin in Table 2; these being those of Sturkie and Newman (1951) and Wu et al. (2017). These reported concentrations of, respectively, 2.26 and 4.8 g dL^−1^; these being more than four times the SD above the means of the other values.

Serum/plasma albumin concentrations were similar in sexually immature female chickens and in non-reproductive female and male mallards [respectively, 1.64 ± (26) 0.08 and 1.83 ± (53) 0.03 g dL^−1^ calculated from Driver, 1981; Fairbrother et al., 1990]. In contrast, plasma/serum concentrations of albumin were increased 25.7% (*p* < 0.001) between not laying and laying females [respectively 1.83 ± (53) 0.03 g dL^−1^; 2.30 ± (18) 0.08 g dL^−1^ calculated from Fairbrother et al., 1990].

#### Interstitial Fluid Protein Concentrations

Based on suction blister fluid, the protein concentrations in interstitial fluid of chickens have been reported (Table 3). The concentrations of albumin, α-globulin, β-globulin, and γ-globulin in interstitial fluid are less than a half that of serum with the greatest decline being with α_2_-globulin.

**Table 3:** Comparison of concentrations of yolk constituents with their concentrations in plasma/serum and egg yolk in chickens.

Plasma/serum equivalent	Yolk component	Plasma/serum g dL^−1^	Egg yolk g dL^−1^	Ratio plasma/serum: yolk	Interstitial fluid g dL^−1^	Ratio Interstitial fluid: yolk
Albumin	α-livetin	1.67^P^	1.06^Q^^R^	0.61	0.89^S^	1.19
α_2_-glycoprotein	β-livetin	0.268^S^	2.65^Q^^R^	**9.89**	0.11^S^	**24.1**
γ-globulin (IgY)	γ-livetin	0.948^P^	1.59^Q^^R^	1.68	0.37	**4.54**
Vitellogenin	Phosvitin + lipovitellin	1.63^P^	10.4^Q^	**6.38**	NA	NA
Triglyceride	Triglyceride	2.268^P^	25.1^Q^	**11.1**	NA	NA
Phospholipids	Phospholipids	0.453^P^	5.89^Q^	**13.0**	NA	NA

*Bold and underlined indicates ratio of >4.5*.

P *From Table 2*.

Q *Calculated from Gilbert (1971)*.

R *Calculated from Bernardi and Cook (1960), also see reviews e.g., Schade and Chacana (2007) for the ratio of α-livetin: β-livetin: γ-livetin being 2:5:3*.

*NA, data not available.Adapted from Peltonen and Sankari (2011)*.

#### Transfer of Yolk Precursors From the Plasma Into the Oocyte

There is transfer of constituents of yolk from the serum into the oocyte in the process of yolk deposition. Table 3 summarizes concentrations of yolk precursors in plasma/serum with constituents of yolk. There is clearly considerable bio-concentration of the following: vitellogenin, α_2_-glycoprotein/β-livetin, triglyceride, and phospholipids.

### Organs

#### Relative Organ Weights in Pullets and Laying Hens

There were no differences between sexually immature female chickens (pullets) and laying hens in the relative weights of liver, gizzard, kidneys, and spleen (Table 4; for details of different studies, see Supplementary Table 7). In contrast, as might be expected, the relative weights of the ovary and oviduct were markedly greater (*p* < 0.01) in laying hens than in sexually immature female chickens; the increases being, respectively, 52.6 and 60.9 fold for the ovary and oviduct (Table 5). It is noted that there is a single ovary and that all the entire female reproductive tract is referred to as the oviduct (for details of different studies species see Supplementary Table 7).

**Table 4:** Differences in reproductive and other parameters between sexually immature female chickens and laying hens.

	Mean ± (*n* = studies) SEM	
	Immature females	Laying hens
Relative weights g 100 g^−1^		
Gizzard	3.06 ± (11) 0.58^A^^P^	3.21 ± (7) 1.10^A^^P^
Kidneys	0.65 ± (8) 0.14^A^	0.80 ± (5) 0.098^A^
Liver	2.04 ± (12) 0.19^A^	2.33 ± (23) 0.10^A^
Small intestine	4.15 ± (3) 0.87^A^^Q^	3.39 ± (3) 1.22^A^^Q^
Spleen	0.176 ± (8) 0.025^A^	0.160 ± (8) 0.022^A^
Ovary	0.046 ± (4) 0.015^A^	2.42 ± (15) 0.19^A^^***^
Oviduct	0.046 ± (2) 0.024^A^	2.80 ± (9) 0.20^A^^**^
Liver cellularity		
Liver cell number × 10^9^ per g	1.417 ± (4) 0.192^B^	1.127 ± (4) 0.113^*^^B^
Liver cell volume fL	526 ± (4) 112^C^	682 ± (4) 138^*^^C^
Liver composition		
Lipid g 100 g^−1^	4.4 ± (4) 0.75^D^^E^	13.0 ± (3) 1.54^**^^F^

*Difference between laying hens and immature chickens*.

* *p < 0.05*.

** *p < 0.01*.

*** *p < 0.001*.

A *Calculated from data in Supplementary Tables 6, 7*.

B *Calculated from DNA per gram liver and assumes liver is 90% cells includes laying hens and estradiol treated young females*.

C *Calculated from avian DNA per g liver data (Common et al., 1951; Jost et al., 1973; Yu and Marquardt, 1973; Neill et al., 1977) and female chickens having 2.15 pg DNA per cell (Mendonça et al., 2010, 2016)*.

D *Based on sexually immature female and male chickens*.

E *Lorenz et al. (1938); Trampel et al. (2005); Seong et al. (2015); and ARS (United States Department of Agriculture Agricultural Research Service) (2018)*.

F *Lorenz et al. (1938); Naber and Biggert (1989); and Cherian and Goeger (2004)*.

P *In comparison, the mean relative weight of the gizzard in broiler chickens was 2.932 ± (number of studies = 6) SEM 0.64 (for data on individual studies, see Supplementary Table 7A)*.

Q *In comparison, the mean relative weight of the small intestine in broiler chickens was 3.59 ± (7) 0.36 (for data on individual studies, see Supplementary Table 7A)*.

**Table 5:** Small intestine parameters in laying hens [mean ± (number of studies) SEM].

	Duodenum	Jejunum	Ileum
Length			
Chicken cm^P^	26.6 ± (10) 2.1	59.3 ± (9) 6.0	42.7 ± (10) 4.6
Laying hen (cm kg^-1^)^R^	15.7	33.7	33.9
Mucosa^S^			
Thickness μm^S^	2,388	1,877	1,111
DNA mg g^-1^^T^	3.23	2.74	NA
Cellularity # g^-1^^V^	1.474 × 10^9^	1.251 × 10^9^	NA
Cell mass in ng or volume in pl	0.678	0.799	NA
Villus			
Height μm^W^	1,357 ± (19) 109.9	960 ± (23) 59.0	721 ± (20) 59.9
Width μm^W^	276.8 ± (8) 74.9	148.1 ± (8) 21.2	216.2 ± (7) 59.3
Villus amplification factor	11.62	9.04	7.93
Microvillus			
Length μm^X^	1.463	1.467	1.029
Diameter μm^X^	0.0705	0.0705	0.0787
Density # μm^2^^X^	113.0	76.8	104.1
Microvillus amplification factor	37.6^X^	38.8^Y^	27.5^X^

P *Calculated from data in Supplementary Table 8*.

R *Ding et al. (2018)*.

S *Shang et al. (2015)*.

T *Hu and Guo (2007)*.

V *Calculated based on 2.19 pg DNA per cell in chickens (Mendonça et al., 2010, 2016)*.

W *Calculated from data in Supplementary Table 9*.

X *Ferrer et al. (1995)*.

Y *Jejunum: 35.9 (Ferrer et al., 1991); 41.7 (Mitjans et al., 1997)*.

*NA, not available*.

#### Cellularity of the Liver and Mucosa

The tendency for relative weight to be greater in laying hens was matched by an increase in the liver cell volume as calculated from the hepatic DNA concentration (Table 4). The calculated volume of liver cells was similar to that for mucosal cells (Table 5).

#### Bile Production

There was no difference in the rate of bile production in chickens vs. mammals (Table 6).

**Table 6:** Gastro-intestinal and accessory organ parameters in chickens compared to those in mallards and mammals.

Gastro-intestinal parameters	Mean ± (studies) SEM		
	Chicken	Mallards/domestic ducks	Mammalian species for comparison
Bile production mL h^−1^ kg b.w.^-1^^A^	1.157 ± (6) 0.613	NA	2.034 ± (9) 0.604
Villus Height μm			
Duodenum^B^^,^^C^	1,357 ± (19) 109.9	891 ± (5) 111.7^*^	574 ± (9) 61.7^#^
Jejunum^B^^,^^C^	960 ± (23) 59.0	862 ± (3) 41.1	529 ± (10) 58.4^#^
Ileum^B^^,^^C^	721 ± (20) 59.9	747 ± (3) 77.4	370 ± (9) 53.7^#^
Absorptive area of small intestine			
m^2^ (cm^2^ × 10^4^)	7.78^D^	5.67^E^	15.5 ± (8) 5.4^F^
m^2^ kg^−1^	4.86^D^	7.80^E^	18.7 ± (8) 6.8^F^

*NA, Not available*.

* *Different from chicken *p* < 0.05*.

# *Different from chicken and mallard *p* < 0.05*.

A *Based on data in Supplementary Table 13 of Supplementary Data*.

B *Data on different studies with mallards and domestic ducks are available in Supplementary Table 10 of Supplementary Data*.

C *Data on different mammals is available in Supplementary Table 11*.

D *Calculated from Ferrer et al. (1995) and Mitjans et al. (1997)*.

E *Watkins et al. (2004)*.

F *Based on bat species 1 (absorptive area 5.42 cm^2^ × 10^4^, 44.4 m^2^ kg^−1^), bat species 2 (absorptive area 29.4 cm^2^ × 10^4^, 47.1 m^2^ kg^−1^), rat (absorptive area 1.8 cm^2^ × 10^4^, 4.85 m^2^ kg^−1^), rabbit (absorptive area 12.1 cm^2^ × 10^4^, 5.48 m^2^ kg^−1^), dog (absorptive area 39.9 cm^2^ × 10^4^, 3.41 m^2^ kg^−1^), desert rat (absorptive area 4.1 cm^2^ × 10^4^, 12.2 m^2^ kg^−1^; Ferraris et al., 1989), mouse (absorptive area 1.10 cm^2^ × 10^4^, 31.5 m^2^ kg^−1^; Ferraris et al., 1989; Casteleyn et al., 2010), and human (absorptive area 30 cm^2^ × 10^4^, 0.48 m^2^ kg^−1^; Helander and Fändriks, 2014)*.

#### Small Intestine Length

It is predicted that the length and other parameters of the small intestine would increase during growth. Under these circumstances, length of the small intestine would be expected to be greater in laying hens than in young broiler chickens. This was not the case. Small intestinal lengths were essentially identical (*p* > 0.05) between young broiler chickens and laying hen (>20 week-old) [young 3–6 week—old broiler chickens: 131.8 ± (*n* = 5 studies) SEM 22.3 cm vs. laying hens: 129.4 ± (4) 7.4 cm]. Under the above circumstances, small intestinal parameters are shown for chickens irrespective of age (>3 week-old) or reproductive status (Table 5). The length of the caeca and colon/rectum in chickens were, respectively, 28.1 ± (5 studies) 3.9 and 9.48 ± (5) 0.72 cm in length.

The length of the small intestine and its components in laying hens (Table 5) are very similar to those in mallards/domestic ducks (mallard—small intestine: 81.4 cm kg^−1^; duodenum: 17.1 cm kg^−1^; jejunum: 31.5 cm kg^−1^; and ileum 32.8 cm kg^−1^; Kokoszyński et al., 2017).

There was no relationship between the length of the small intestine in broiler chickens and their age [length vs. age of broiler chickens adjusted *R*^2^ 0.0392 (*p* = 0.360)]. This is in contrast to the relationships between small intestinal length and body weight across multiple avian species [small intestine length vs. body weight: adjusted *R*^2^ 0.805 (*p* = 05.141E^−12^), slope 0.00454 ± (31 species) 0.00041; log_10_ small intestine length vs. log_10_ body weight (allometric relationship): adjusted *R*^2^ 0.692 (*p* = 4.021E^−9^), slope 0.344 ± (31) 0.042; relative small intestine length (as percentage of body weight) vs. log_10_ body weight: adjusted *R*^2^ 0.707 (*p* = 1.980E^−9^), slope − 36.6 ± (31) 4.28; for details of species see Supplementary Table 11].

#### Small Intestine Villi Morphological Characteristics

As might be expected and based on studies where the villus height/length was determined in the three regions of the small intestine, the villus height/length was greater in the duodenum than either the jejunum (*p* < 0.05) or ileum (*p* < 0.01). The villus length/height was markedly higher (*p* < 0.01) in chickens than in mammals; the increases being the following: duodenum 136.3%, jejunum 81.4%, and ileum 94.5% (see Table 6).

Villus heights/lengths were identical (*p* > 0.05) between young broiler chickens and laying hen [>20 week-old; duodenum: young broiler chickens: 1,440 ± (14 studies) 135.4 μm vs. laying hens: 1,394 ± (2) 85.5 μm; jejunum: young broiler chickens: 989 ± (15) 78.9 μm vs. laying hens: 985 ± (4) 73.7 μm; Table 6; for details of studies, see Supplementary Table 9].

#### Small Intestine Microvilli Morphological Characteristics

There is no information in the literature on the characteristics of microvilli in laying hens. Therefore, data from young chickens had to be employed. The microvilli were approximately 1.0–1.5 μm long (Table 6) with the mean in four studies being 2.07 ± (4) 0.27 μm (Ferrer et al., 1995; Mitjans et al., 1997; Fischer da Silva et al., 2007). Similarly, the microvilli are present in the ceca and colon [Ceca: length 0.582 μm, diameter 0.089 μm; density 89.9 # μm^−2^; colon: length 0.597 μm, diameter 0.079 μm; and density 86.7 # μm^−2^; Ferrer et al., 1995].

#### Gastrointestinal Absorptive Area

The absorptive areas and absorptive areas per kg body weight in chickens and mallard duck have been calculated (see Table 6). These fall within the range for mammalian species (see Table 6).

## Discussion

The present report provides a comprehensive compendium of physiological and anatomical characteristics of two species important to avian toxicological research and to addressing regulatory requirements; namely the laying hen (adult sexually mature female chicken—*Gallus gallus*) and mallard duck (*Anas platyrhynchos*). Moreover, such accounting of the physiological and anatomical characteristics of laying hens and mallard ducks is essential to the development of PBK models. When there is a need to extrapolate parameterization from the chicken (laying hen) to the mallard duck when species specific data are unavailable, it is recommended that consideration be given to using data from sexually immature female chicken rather than laying hens.

### Fluid Dynamics in Adult Female Chickens or Ducks

Fluid dynamics in laying hens and mallards (Table 1) are key information for the development of PBK models. Cardiac output is decreased in starved chickens (Vogel and Sturkie, 1963). It is, therefore, possible that chemical agents that decrease voluntary feed intake will also depress cardiac output.

### Blood

Both the Hct/PCV and hemoglobin of mallards (Table 1) are similar to those across avian species [Hct/PCV 44.0% based 158 species of birds; hemoglobin 14.5 g dL^−1^ based 134 species of birds; Scanes, 2022]. In contrast, both the Hct/PCV of laying hens (Tables 1, 2) are markedly lower than that in mallards and the mean across avian species. Blood concentrations of hemoglobin are similarly low in reptiles [Crocodilia: 8.23 g dL^−1^ (three species); Dessauer, 1970, 7.76 g dL^−1^ (five species); Pough, 1980; Squamata: 7.77 g dL^−1^; Dessauer, 1970, 8.8 g dL^−1^ (Pough, 1980); and Testudines: 7.97 g dL^−1^; Dessauer, 1970, 8.5 g dL^−1^; Pough, 1980]. The last common ancestor being the following:

- Squamata and Testudines/Crocodilia/Aves 255 Million Years Ago (MYA)
- Crocodilia/Aves and Testudines 250 MYA (Chiari et al., 2012; Ezcurra et al., 2014).

Laying hens were found to exhibit a lower Hct/PCV and hemoglobin than mallards (Table 1). This reduction in the Hct/PCV and hemoglobin concentrations would reduce the number of available binding sites for chemical agents, a finding that is likely to impact PBK modeling.

Serum/plasma concentrations of total protein and albumin are very similar in laying hens (Table 2), in mallards and across birds (total protein concentration: 39.6 across 100 avian species; 15.9 across 63 avian species; Scanes, 2022). The increase in total protein in the serum/plasma in mallards between reproductively quiescent and laying females is presumed to reflect the appearance of vitellogenin in the circulation of laying ducks.

### Implications of Low Albumin Concentrations in Laying Hen and Mallards

There are at least three binding sites on human albumin, respectively, the following:

- Site 1: the “warfarin” binding site
- Site 2: the “benzodiazepine” binding site
- Site 3: the “deltamethrin” binding site

It is reasonable to assume that avian albumin has analogous binding sites. The presence of such binding sites is likely to impact PBK modelling as is the concentration of albumin in plasma/serum (Sjöholm et al., 1979; Sethi et al., 2016; also see review: Yang et al., 2014).

Plasma/serum concentration of albumin in laying hens (Table 2) and laying female mallards (discussed above) are lower than in mammals [3.59 ± (39) 0.09 g dL^−1^; Scanes, unpublished]. This has implications for PBK modeling, as there are likely to be increases in the fraction unbound of compound for specific drugs or pesticides (Sethi et al., 2016). For instance, the percentage of unbound compound was much higher in the plasma of human neonates (diazepine: 15.4%, cyclosporine 20.0%, and deltamethrin 26.9%) than in adults (diazepine: 4.1%, cyclosporine 4.3%, and deltamethrin 10.0%); there being higher plasma concentrations of albumin in adults (2.8 g dL^−1^) vs. neonates (4.0 g dL^−1^).

There is a strong case for determining the unbound fraction for chemical agents in the following: (1) hen plasma, (2) immature female chicken plasma, (3) purified chicken serum albumin, and (4) vitellogenin. It is suggested that studies could employ heparinized blood from laying hens and young female chickens to determine in distribution between plasma and erythrocytes and within plasma the free unbound.

### Bio-Concentration of Precursors During Yolk Deposition Into the Oocyte

It is interesting that there is bio-concentration of vitellogenin, α_2_-glycoprotein/β-livetin, triglyceride, and phospholipids into yolk but not of albumin or immunoglobulin compared to plasma (Table 3). There is also bio-concentration of α_2_-glycoprotein/β-livetin and immunoglobulin compared to interstitial fluid (Table 3). The basis for the bioconcentration is receptor-mediated transport. A specific receptor has been identified that mediates transfer of both vitellogenin (Vt) and very-low-density lipoprotein (VLDL), including triglyceride and phospholipid, across the oocyte plasma membrane to fill the oocyte with yolk (chickens: Stifani et al., 1990; Barber et al., 1991; Bujo et al., 1994). Furthermore, the receptor binds α_2_-glycoprotein (Jacobsen et al., 1995). There are, in addition, other receptors mediating transfer into the oocyte. What is not known is the extent to which chemical agents are transferred into the oocyte bound (or loosely associated) with Vt or VLDL and thereby pass into the oocyte by receptor-mediated transport. This would not be consistent with a simplistic view in which only “fraction unbound” chemical agents can pass into cells by passive diffusion.

Chemical agents can bind to yolk precursors in the blood and, thereby, be deposited into the oocyte (yolk). In older literature, radiolanthanum has been demonstrated to bind to yolk proteins (granules and specifically phosvitin) both *in vivo* and *in vitro* (Robinson et al., 1979). Deposition into the yolk has been examined in Japanese quail. Radiolanthanum and other radiolanthanides were administered intravenously (Robinson et al., 1980). After 18 h, 15% of the dose was located in the oocytes with ~90% of the dose being located in the eggs after 10 days (Robinson et al., 1978, 1980). Similar results were reported for other radiolanthanides (Robinson et al., 1980). This is consistent with radiolanthanides binding to the phosvitin component of vitellogenin and then receptor mediated transport across the oocyte membrane.

### Organ Parameters

As might be expected, ovary and oviduct masses were much greater in laying hens than in sexually immature chickens (Table 4). In contrast, there were no differences in the relative weights of the gizzard, heart, kidneys, liver, small intestine, and spleen between those in sexually immature chickens and in laying hens (Table 4). However, as female chickens come into lay there is a 29.2% increase in body weight (Hurwitz and Bar, 1971). Thus, conclusions based on relative weights of the gizzard, kidneys, liver, and spleen (Table 4) are confounded by the increase in body weights.

### Liver

Like its mammalian counterparts, the avian liver is critically important for controlling metabolism and, for many toxicants, detoxification (Zaefarian et al., 2019). The avian liver receives blood *via* both the hepatic artery and the hepatic portal vein (Zaefarian et al., 2019). The liver produces bile. In Galliform and Anseriform birds such as chickens and mallard ducks, the major bile acid is chenodeoxycholic acid (Hagey et al., 2010a) while in mammals, the major bile acids are cholic acid together with chenodeoxycholic acid (Hagey et al., 2010b). In addition, 23 and 15α-hydroxylated forms are found in both birds and mammals (Hagey et al., 2010b). Chemical agents can be eliminated in the bile but there is recycling with a chemical agent or its metabolite passing from the blood through the liver into the bile. The bile passes to the small intestine where the chemical agent or its metabolite is (re-)absorbed into the blood. It is suggested that the avian liver affects detoxification and excretion of chemical agents in a manner similar to that in mammals and that PBK modelling can use parameters developed for the mammalian liver.

### Kidney

There are some differences between the avian and mammalian kidneys. A renal portal system is present in birds including chickens and mallards but not mammals (reviewed: Akester, 1967). Closure of the left and/or right renal portal valves diverts blood from the left/right external iliac veins to renal portal veins in chickens, mallards, and other avian species (Akester, 1967). Blood flow can also be diverted from the kidney to the central circulation (vena cava; Burrows et al., 1983). Blood flow in the coccygeo-mesenteric vein can flow in either direction allowing blood flow into the hepatic portal system (Akester, 1967). The valves in the renal portal system are controlled by the sympathetic nervous system (Burrows et al., 1983).

It is reasonable to conclude that there is renal intestinal recycling of toxicants in mallards and chickens. Recycling encompasses a chemical agent or its metabolite passing from the blood, filtered into nephrons, and hence into the urine. This passes from the cloaca to the colon and caeca by retrograde peristalsis and then is absorbed into the blood stream.

### Gastro-Intestinal Tract

There are marked similarities between the gastrointestinal tract between mammals and the laying hen or mallards with an esophagus, the equivalent of a stomach, a small intestine, and a colon. There are several major differences between the anatomy of the gastrointestinal tract of birds compared to mammals including the presence of the following: (1) A sac-like organ off the esophagus—the crop; (2) A separate glandular and muscular stomach, namely, the proventriculus and gizzard; (3) Two ceca; and (4) The absence of teeth.

Absorption and reabsorption of nutrients or chemical agents predominantly occur in the small intestine. In the present analysis, the absorptive areas in chickens and mallard ducks were very similar (Table 6). Moreover, they were with the range for mammals (Table 6). This suggests that the characteristics of absorption of chemical agents in these avian species is similar to that in mammals, although the specificity and kinetics of active transport in birds needs to be included in any analysis.

The lack of a difference between jejunal villus height in young broiler chickens and adult laying hens (Table 5) is in contrast to a relationship, albeit modest (*R*^2^ 0.24) between log villus height and log body weight across multiple avian species (Ricklefs, 1996).

Absorption and reabsorption of chemical agents in the urine may also occur in the colon and ceca due to retrograde flow and colonic anti-peristalsis (Lai and Duke, 1978; Duke, 1989). It has been demonstrated that drugs can be administered as an enema in birds (day-old chicks: Marietto-Gonçalves). Moreover, rehydration can be accomplished by rectal administration of a hypotonic solution (pigeons: Ephrati and Lumeij, 1997) supporting both retrograde peristalsis and absorption in the colon and perhaps also the ceca.

### Comparison of Parameters in the Present Study and Others Studies

There is broadly both very similar numerical values between the preset study together with complementarity between the present analyses and those of Wang et al. (2020) with multiple parameters reported in one but the other (Tables 7–9). The former was despite the greater number of studies employed in the present analysis (Table 7). An exception to this is the markedly greater relative weights of the gizzard and small intestine in the present report and that of Wang et al. (2020) (Table 7). This difference may reflect the greater number of studies employed in the present analysis together with the extremely low relative organ weights in the study of Martínez et al. (2015). Moreover, in the present meta-analysis, there were similar relative weights of the gizzard and small intestine in sexually immature females and laying hens (Table 4; Wang et al., 2020).

**Table 7:** Comparison of organ weights, cardiac output and hematocrit in laying hens and those reported by Wang et al. (2020).

Parameter organ	Mean in laying hens ± (number of studies)	
	Present study ± SEM	Wang et al. (2020)^Δ^ ± SD
Relative organ weight (Organ weight as % b.wt.)		
Blood	6.3 (1)	6.3 ± (1) 1.33^Ψ^
Heart	0.45 ± (9) 0.040	0.30 ± (1) 0.065
Liver	2.33 ± (23) 0.10	2.49 ± (2) 0.49
Kidneys	0.80 ± (5) 0.098	0.76 ± (2) 0.13
Spleen	0.16 ± (8) 0.022	0.15 ± (3) 0.06
Gizzard	3.21 ± (7) 1.10	0.70 ± (2) 0.11^A^^B^
Small intestine	3.39 ± (3) 1.22	0.95 ± (1)^B^
Ovary	2.38 ± (14) 0.21	1.91 ± (1) 0.28
Oviduct	2.84 ± (8) 0.22	2.58 ± (3)
Cardiovascular		
Cardiac output L hr.^−1^ kg b.wt.^-1^^A^	10.6 (7)^B^	9.91 ± (6)^Λ^
Hematocrit	27.1 (10)^Γ^	31.0 ± (5)

*Bold indicates a marked difference between the present study and that of Wang et al. (2020)*.

A *For individual study means, see Supplementary Table 1*.

B *Study of laying hens by Wolfenson et al. (1981) (gizzard relative weight 0.98 g 100 g b.wt.^−1^, small intestine relative weight 0.95 g 100 g b.wt.^−1^)*.

Λ *Study of pullets, not laying hens, by Martínez et al. (2015) (gizzard relative weight 0.333 g 100 g b.wt.^−1^, small intestine relative weight 0.260 g 100 g b.wt.^−1^)*.

Ψ In contrast, in Table 24 of Wang et al. (2020), blood is listed as 2.36% of body weight in laying hens. There was no explanation for the disparity.

Δ Based on Table 3 in Wang et al. (2020).

**Table 8:** Comparison of blood flow in laying hens and those reported by Wang et al. (2020).

Organ	Blood flow mL min^-1^^Δ^^,^^Φ^^,^^Ω^^,^^A^	
	Present report	Wang et al. (2020)
Cerebrum^Ψ^	NA	0.016 (1)
Cerebellum^Ψ^	NA	0.0027 (1)
Proventriculus	{5.8 ♂^3^}	2.93 (4)
Gizzard	8.0 (1)^1^	2.24 (4)
Duodenum	15.3^2^	12.5 (5)
Jejunum	NR	12.3 (5)
Ileum	NR	5.6 (1)
Colon	NR	0.32 (2)
Heart	20.6 ± (2) 2.2^1^^,^^2^	14.4 (1)
Liver arterial	26.9 ± (2) 6.4^1^^,^^2^	66.7 (6)
Liver portal	0.88	41.6 (2)
Muscle pectoralis	5.3 (1)^1^	20.0 (2)
Lungs^Φ^	NA	149.1 (2)
Kidneys	52.3 ± (2) 20.6^1^^,^^2^	53.1 (6)
Spleen	6.59 ± (2) 4.11^1^^,^^2^	10.7 (1)
Ovary	**8.79** ± **(5) 3.06**^B^	**43.5 (1)**
Oviduct	27.0+ (8) 5.45	32.3 (1)
Infundibulum	0.95 ± (6) 0.056^B^	0.83 (5)
Magnum	19.3 ± (7) 2.11^B^	13.6 (6)
Isthmus	2.34 ± (7) 0.36^B^	3.2 (5)
Uterus or shell gland	9.78 ± (7) 2.07^B^	14.7 (5)
Vagina	1.00 ± (3) 0.12^B^	1.07 (2)

*Bold indicates marked difference between present study and that of Wang et al., 2020*.

*NA, not applicable based on the microsphere methodology*.

*NR, not reported*.

1 *Sapirstein and Hartman (1959)*.

2 *Boelkins et al. (1973)*.

3 *Merrill et al. (1981)*.

A *Reported in most literature as mL min^−1^ kg^−1^*.

B *For details in individual studies see Supplementary Table 14*.

Δ *It is unclear why blood flow is per kg b.wt. and not per organ weight*.

Φ *The reported values pool all ages of chickens predominantly broiler chicken (males and females), laying hens, and in one case adult male chickens except for the ovary and oviduct that are limited to laying hens*.

Ω *The sum of blood flow is 18.9 L hr.^−1^ kg m.wt.^−1^. This is markedly higher than cardiac output. In addition, some organs are not included such as bone, ovary, and oviduct*.

Ψ *There are methodological problems with blood flow to the brain and lungs*.

**Table 9:** Comparison of data included in the present report and that of Wang et al. (2020).

	This report	Wang et al. (2020)	Gastrointestinal characteristics continued	This report	Wang et al. (2020)
Total body water % of b.wt.		X	Weight		
Intracellular water % of b.wt.	✓	X	Duodenum	✓	✓
Extracellular water % of b.wt.	✓	X	Jejunum	✓	✓
Plasma % of body weight	✓	✓	Ileum	✓	✓
Interstitial fluid % of b.wt.	✓	X	Length		
Hematocrit/PCV %	✓	✓	Duodenum	✓	X
Hemoglobin %	✓	X	Jejunum	✓	X
Plasma constituents			Ileum	✓	X
Plasma proteins g dL^−1^	✓	X	Mucosal thickness		
Albumin g dL^−1^	✓	X	Duodenum	✓	X
IgY g dL^−1^	✓	X	Jejunum	✓	X
IgA g dL^−1^	✓	X	Ileum	✓	X
IgM g dL^−1^	✓	X	Villus height		
Vitellogenin g dL^−1^	✓	X	Duodenum	✓	X
Triglyceride g dL^−1^	✓	X	Jejunum	✓	X
Cholesterol g dL^−1^	✓	X	Ileum	✓	X
Phospholipidsa	✓	X	Villus width		
Relative organ weights			Duodenum	✓	X
Crop	X	✓	Jejunum	✓	X
Proventriculus	X	✓	Ileum	✓	X
Small intestine	✓	✓	Villus amplification factor		
Duodenum	✓	✓	Duodenum	✓	X
Jejunum	✓	✓	Jejunum	✓	X
Ileum	✓	✓	Ileum	✓	X
Ceca	X	✓	Microvillus length		
Colon	X	✓	Duodenum	✓	X
Oviduct	✓	✓	Jejunum	✓	X
Brain	X	✓	Ileum	✓	X
Gastrointestinal characteristics			Microvillus amplification factor		
Absorptive area			Duodenum	✓	X
Duodenum	✓	X	Jejunum	✓	X
Jejunum	✓	X	Ileum	✓	X
Ileum	✓	X	Bile production	✓	X

Turning to the issue of hematocrits, there was only a small difference in hematocrits (Table 7). There were considerably more replicate studies employed in the present analysis (10 studies) compared to Wang et al. (2020) (five studies). However, this difference is unlikely to influence PBK analysis as the fraction unbound is present in the plasma not the erythrocytes.

Blood flow to the ovary reported in the present report differed from that in Wang et al. (2020). This would indicate a lower reliability of either estimate with this being supported by the high coefficient of variation (Table 8). Moreover, a number of studies were excluded from the present analysis because blood flow to ovarian follicles was only expressed relative to follicular tissue weight (e.g., Hrabia et al., 2005; Rząsa et al., 2008). Unfortunately, these could not be included in the analyses.

Physiological and anatomical data on laying hens and mallards/domestic ducks (*Anas platyrhynchos*) have been rigorously compiled and subjected to analysis. As a comprehensive compendium, these data provide one of the critical bases for the development of PBK models for laying hens and mallards.

## Data Availability Statement

The original contributions presented in the study are included in the article/**Supplementary Material**; further inquiries can be directed to the corresponding author.

## Author Contributions

CS, JW, ME, SS, VB, AB, TP, and DH conceived the study, critiqued analysis, and revised the manuscript. CS and VB collected data. CS conducted the analyses and drafted the manuscript. All authors contributed to the article and approved the submitted version.

## Funding

The authors declare that this study received funding from Bayer AG. As stated in the author contributions, the funder (in the person of the Bayer authors) had the following involvement with the study: contributed to conceiving the study, revising the manuscript, and critiquing the analysis.

## Conflict of Interest

JW, ME, AB, TP, and DH were employed by the company Bayer AG. SS and VB were employed by the company esqLABS GmbH. CS, his company Scanes Technology and Research and esqLABS GmbH received funding from Bayer AG.

## Publisher’s Note

All claims expressed in this article are solely those of the authors and do not necessarily represent those of their affiliated organizations, or those of the publisher, the editors and the reviewers. Any product that may be evaluated in this article, or claim that may be made by its manufacturer, is not guaranteed or endorsed by the publisher.

## References

[ref1] Akester A. R. (1967). Renal portal shunts in the kidney of the domestic fowl. J. Anat. 101, 569–594. PMID: 6051735 PMC1270933

[ref2] ARS (United States Department of Agriculture Agricultural Research Service). (2018). National Nutrient Database for Standard Reference. Available at: https://ndb.nal.usda.gov/ndb/ (Accessed August 13, 2021).

[ref3] Barber D. L. Sanders E. J. Aebersold R. Schneider W. J. (1991). The receptor for yolk lipoprotein deposition in the chicken oocyte. J. Biol. Chem. 266, 18761–18770. doi: 10.1016/S0021-9258(18)55128-0, PMID: 1655760

[ref5] Bernardi G. Cook W. H. (1960). Separation and characterization of the two high-density lipoproteins of egg yolk, α- and β-lipovitellin. Biochim. Biophys. Acta 44, 96–105. doi: 10.1016/0006-3002(60)91527-4

[ref6] Boelkins J. N. Mueller W. J. Hall K. L. (1973). Cardiac output distribution in the laying hen during shell formation. Comp. Biochem. Physiol. A 46, 735–743. doi: 10.1016/0300-9629(73)90125-4, PMID: 4127752

[ref7] Bujo H. Hermann M. Kaderli M. O. Jacobsen L. Sugawara S. Nimpf J.. (1994). Chicken oocyte growth is mediated by an eight ligand binding repeat member of the LDL receptor family. EMBO J. 13, 5165–5175. PMID: 7957081 10.1002/j.1460-2075.1994.tb06847.x PMC395465

[ref8] Burrows M. E. Braun E. J. Duckles S. P. (1983). Avian renal portal valve: a reexamination of its innervation. Am. J. Phys. 245, H628–H634. doi: 10.1152/ajpheart.1983.245.4.H628, PMID: 6624932

[ref9] Casteleyn C. Rekecki A. Van der Aa A. Simoens P. Van den Broeck W. (2010). Surface area assessment of the murine intestinal tract as a prerequisite for oral dose translation from mouse to man. Lab. Anim. 44, 176–183. doi: 10.1258/la.2009.009112, PMID: 20007641

[ref11] Cherian G. Goeger M. P. (2004). Hepatic lipid characteristics and histopathology of laying hens fed CLA or n-3 fatty acids. Lipids 39, 31–36. doi: 10.1007/s11745-004-1198-2, PMID: 15055232

[ref12] Chiari Y. Cahais V. Galtier N. Delsuc F. (2012). Phylogenomic analyses support the position of turtles as the sister group of birds and crocodiles (Archosauria). BMC Biol. 10:65. doi: 10.1186/1741-7007-10-65, PMID: 22839781 PMC3473239

[ref13] Common R. H. Chapman D. G. Maw W. A. (1951). The effect of gonadal hormones on the nucleic acid content of liver and serum in the immature pullet, and the difference between the nucleic acid content of the livers of sexually mature pullets and cockerels. Can. J. Zool. 29, 265–275. doi: 10.1139/z51-024

[ref14] Cortright K. A. Wetzlich S. E. Craigmill A. L. (2009). A PBPK model for midazolam in four avian species. J. Vet. Pharmacol. Ther. 32, 552–565. doi: 10.1111/j.1365-2885.2009.01073.x, PMID: 20444010

[ref15] de Montaigu T. Goulson D. (2020). Identifying agricultural pesticides that may pose a risk for birds. Peer 8:e9526. doi: 10.1111/j.1365-2885.2009.01073.x, PMID: 32832262 PMC7413080

[ref16] Dessauer H. C. (1970). “Blood chemistry of reptiles: physiological and evolutionary aspects,” in Biology of Reptiles. Vol. 3. eds. Gans C. Parsons T. S. (New York: Academic Press), 1–72.

[ref17] Ding X. M. Li D. D. Bai S. P. Wang J. P. Zeng Q. F. Su Z. W.. (2018). Effect of dietary xylooligosaccharides on intestinal characteristics, gut microbiota, cecal short-chain fatty acids, and plasma immune parameters of laying hens. Poult. Sci. 97, 874–881. doi: 10.3382/ps/pex372, PMID: 29294100

[ref18] Driver E. A. (1981). Hematological and blood chemical values of mallard, Anas p. platyrhynchos, drakes before, during and after remige moult. J. Wildl. Dis. 17, 413–421. doi: 10.7589/0090-3558-17.3.413, PMID: 7310951

[ref19] Duke G. E. (1989). Relationship of cecal and colonic motility to diet, habitat, and cecal anatomy in several avian species. J. Exp. Zool. Suppl. 252, 38–47. doi: 10.1002/jez.1402520507, PMID: 2575126

[ref20] Ephrati C. Lumeij J. T. (1997). Rectal fluid therapy in birds: an experimental study. J. Avian Med. Surg. 11, 4–6.

[ref23] Ezcurra M. D. Scheyer T. M. Butler R. J. (2014). The origin and early evolution of Sauria: reassessing the Permian saurian fossil record and the timing of the crocodile-lizard divergence. PLoS One 9:e89165. doi: 10.1371/journal.pone.0089165, PMID: 24586565 PMC3937355

[ref24] Fairbrother A. Craig M. Walker K. O’Loughlin D. (1990). Changes in mallard (*Anas platyrhynchos*) serum chemistry due to age, sex and reproductive condition. J. Wildl. Dis. 26, 67–77. doi: 10.7589/0090-3558-26.1.67, PMID: 2304202

[ref25] Ferraris R. P. Lee P. P. Diamond J. M. (1989). Origin of regional and species differences in intestinal glucose uptake. Am. J. Phys. 257, G689–G697. doi: 10.1152/ajpgi.1989.257.5.G689, PMID: 2596605

[ref26] Ferrer R. Planas J. M. Durfort M. Moreto M. (1991). Morphological study of the caecal epithelium of the chicken (*Gallus gallus domesticus* L.). Br. Poult. Sci. 32, 679–691. doi: 10.1080/00071669108417394, PMID: 1933442

[ref27] Ferrer R. Planas J. M. Moretó M. (1995). Cell apical surface area in enterocytes from chicken small and large intestine during development. Poult. Sci. 74, 1995–2002. doi: 10.3382/ps.0741995, PMID: 8825590

[ref28] Fischer da Silva A. V. Maiorka A. Borges S. A. Santin E. Boleli I. C. Macari M. (2007). Surface area of the tip of the enterocytes in small intestine mucosa of broilers submitted to early feed restriction and supplemented with glutamine. Int. J. Poult. Sci. 6, 31–35. doi: 10.3923/ijps.2007.31.35

[ref29] Gilbert A. B. (1971). “The egg: its physical and chemical aspects,” in Physiology and Biochemistry of the Domestic Fowl. Vol. 3. eds. Bell D. J. Freeman B. M. (London: Academic Press), 1378–1399.

[ref30] Goetting V. Lee K. A. Tell L. A. (2011). Pharmacokinetics of veterinary drugs in laying hens and residues in eggs: a review of the literature. J. Vet. Pharmacol. Therap. 34, 521–556. doi: 10.1111/j.1365-2885.2011.01287.x, PMID: 21679196

[ref31] Hagan R. C. Leszczynski D. E. Kummerow F. A. (1984). Comparative plasma lipid response of pullets and laying hens to estradiol and progesterone. Toxicol. Appl. Pharmacol. 76, 483–489. doi: 10.1016/0041-008x(84)90352-1, PMID: 6506074

[ref32] Hagey L. R. Vidal N. Hofmann A. F. Krasowski M. D. (2010a). Complex evolution of bile salts in birds. Auk 127, 820–831. doi: 10.1525/auk.2010.09155, PMID: 21113274 PMC2990222

[ref33] Hagey L. R. Vidal N. Hofmann A. F. Krasowski M. D. (2010b). Evolutionary diversity of bile salts in reptiles and mammals, including analysis of ancient human and extinct giant ground sloth coprolites. BMC Evol. Biol. 10:133. doi: 10.1186/1471-2148-10-133, PMID: 20444292 PMC2886068

[ref34] Helander H. F. Fändriks L. (2014). Surface area of the digestive tract—revisited. Scand. J. Gastroenterol. 49, 681–689. doi: 10.3109/00365521.2014.898326, PMID: 24694282

[ref35] Hrabia A. Paczoska-Eliasiewicz H. Niezgoda J. Rząsa J. (2005). Histamine affects blood flow through the reproductive organs of the domestic hen (*Gallus domesticus*). Folia Biol. 53, 209–213. doi: 10.3409/173491605775142864, PMID: 19058546

[ref36] Hu Z. Guo Y. (2007). Effects of dietary sodium butyrate supplementation on the intestinal morphological structure, absorptive function and gut flora in chickens. Anim. Feed Sci. Technol. 132, 240–249. doi: 10.1016/j.anifeedsci.2006.03.017

[ref37] Hurwitz S. Bar A. (1971). The effect of pre-laying mineral nutrition on the development, performance and mineral metabolism of pullets. Poult. Sci. 50, 1044–1055. doi: 10.3382/ps.0501044, PMID: 5106883

[ref38] Jacobsen L. Hermann M. Vieira P. M. Schneider W. J. Nimpf J. (1995). The chicken oocyte receptor for lipoprotein deposition recognizes alpha 2-macroglobulin. J. Biol. Chem. 270, 6468–6475. doi: 10.1074/jbc.270.12.6468, PMID: 7534764

[ref39] Jost J. P. Keller R. Dierks-Ventling C. (1973). Deoxyribonucleic acid and ribonucleic acid synthesis during phosvitin induction by 17beta-estradiol in immature chicks. J. Biol. Chem. 248, 5262–5266. doi: 10.1016/S0021-9258(19)43596-5, PMID: 4797637

[ref40] Keijer E. Butler P. J. (1982). Volumes of the respiratory and circulating systems tufted and mallard ducks. J. Exp. Biol. 101, 213–220. doi: 10.1242/jeb.101.1.213

[ref41] Kokoszyński D. Bernacki Z. Saleh M. Stęczny K. Binkowska M. (2017). Body conformation and internal organs characteristics of different commercial broiler lines. Bras. Cien. 19, 47–52. doi: 10.1590/1806-9061-2016-0262

[ref42] Krapo G. L. (1981). The role of nutrient reserves in mallard reproduction. Auk 98, 29–38.

[ref43] Lai H. C. Duke G. E. (1978). Colonic motility in domestic turkeys. Am J Dig Dis 23, 673–681. doi: 10.1007/BF01072351, PMID: 685934

[ref44] Lautz L. S. Nebbia C. Hoeks S. Oldenkamp R. Hendriks A. J. Ragas A. M. J.. (2020). An open source physiologically based kinetic model for the chicken (*Gallus gallus domesticus*): calibration and validation for the prediction residues in tissues and eggs. Environ. Int. 136:105488. doi: 10.1016/j.envint.2020.105488, PMID: 31991240

[ref45] Lien T.-F. Lu J.-J. Jan D.-F. (2001). Alterations in lipid metabolism between the growing and the laying periods of white Leghorn layers. Asian Australas. J. Anim. Sci. 14, 1460–1464. doi: 10.1080/00071667708416367

[ref47] Lorenz F. W. Chaikoff I. L. Enteman C. (1938). Liver lipids of the laying and non-laying bird. J. Biol. Chem. 123, 577–585. doi: 10.1016/S0021-9258(18)74144-6

[ref48] Lv Z. Xing K. Li G. Liu D. Guo Y. (2018). Dietary genistein alleviates lipid metabolism disorder and inflammatory response in laying hens with fatty liver syndrome. Front. Physiol. 9:1493. doi: 10.3389/fphys.2018.01493, PMID: 30405443 PMC6207982

[ref50] Martínez Y. Carrión Y. Rodríguez R. Valdivié M. Olmo C. Betancur C.. (2015). Growth performance, organ weights and some blood parameters of replacement laying pullets fed with increasing levels of wheat bran. Rev. Bras. Cienc. Avic. 17, 347–354. doi: 10.1590/1516-635X1703347-354

[ref52] Medway W. Kare M. R. (1959). Blood and plasma volume, hematocrit, blood specific gravity and serum protein electrophoresis of the chicken. Poult. Sci. 38, 624–631. doi: 10.3382/ps.0380624

[ref53] Mendonça M. A. C. Carvalho C. R. Clarindo W. R. (2010). DNA content differences between male and female chicken (*Gallus gallus domesticus*) nuclei and z and w chromosomes resolved by image cytometry. J. Histochem. Cytochem. 58, 229–235. doi: 10.1369/jhc.2009.954727, PMID: 19875846 PMC2825488

[ref54] Mendonça M. A. C. Carvalho C. R. Clarindo W. R. (2016). DNA amount of chicken chromosomes resolved by image cytometry. Caryologia 69, 201–206. doi: 10.1080/00087114.2016.1169086 PMC2825488 19875846

[ref700] Merrill G. F. Russo R. E. Halper J. M. (1981). Cardiac output distribution before and after endotoxin challenge in the rooster. Am. J. Physiol. 241, R67–R71. doi: 10.1152/ajpregu.1981.241.1.R67, PMID: 7018271

[ref55] Mitjans M. Barniol G. Ferrer R. (1997). Mucosal surface area in chicken small intestine during development. Cell Tissue Res. 290, 71–78. doi: 10.1007/s004410050909, PMID: 9377644

[ref56] Morgan E. H. (1975). Plasma iron transport during egg laying and after oestrogen administration in the domestic fowl (*Gallus domesticus*). Quart. J. Exp. Physiol. 60, 233–247. doi: 10.1113/expphysiol.1975.sp002314, PMID: 1041406

[ref57] Naber E. C. Biggert M. D. (1989). Patterns of lipogenesis in laying hens fed a high fat diet containing safflower oil. J. Nutr. 119, 690–695. doi: 10.1093/jn/119.5.690, PMID: 2723816

[ref58] Neill A. R. Reichmann K. G. Conner J. K. (1977). Biochemical, physiological and production indices related to fat metabolism in the laying fowl at various stages of physiological development. Br. Poult. Sci. 18, 315–324. doi: 10.1080/00071667708416367, PMID: 890517

[ref59] Nichols J. W. Bennett R. S. Rossmann R. French J. B. (2010). A physiologically based toxicokinetic model for methylmercury in female American kestrels. Environ. Toxicol. Chem. 29, 1854–1867. doi: 10.1002/etc.241, PMID: 20821642

[ref60] OECD (2021). Guidance Document on the Characterisation, Validation and Reporting of Physiologically Based Kinetic (Pbk) Models for Regulatory Purposes. Series on Testing and Assessment No. 331.

[ref61] Peebles E. D. Burnham M. R. Walzem R. Branton S. L. Gerard P. (2004). Effects of fasting on serum lipids and lipoprotein profiles in the egg-laying hen (*Gallus domesticus*). Comp. Biochem. Physiol. A 138, 305–311. doi: 10.1016/j.cbpb.2004.04.008, PMID: 15313484

[ref63] Peltonen L. M. Sankari S. (2011). Ott’s protein osmotic pressure of serum and interstitial fluid in chickens (*Gallus gallus*): effect of age and gender. J. Exp. Biol. 214, 599–606. doi: 10.1242/jeb.048769, PMID: 21270308

[ref64] Pough F. H. (1980). Blood oxygen transport and delivery. Am. Zool. 20, 173–185. doi: 10.1093/icb/20.1.173

[ref65] Redshaw M. R. Follett B. K. (1976). Physiology of egg yolk production by the fowl: the measurement of circulating levels of vitellogenin employing a specific radioimmunoassay. Comp. Biochem. Physiol. 55, 399–405. doi: 10.1016/0300-9629(76)90068-2, PMID: 9258

[ref66] Rezende M. S. Mundim A. V. Fonseca B. B. Miranda R. L. Oliveira W. Lellis C. G. (2017). Profile of serum metabolites and proteins of broiler breeders in rearing age. Braz. J. Poult. Sci. 19, 583–586. doi: 10.1590/1806-9061-2016-0338

[ref67] Ricklefs R. E. (1996). Morphometry of the digestive tracts of some passerine birds. Condor 98, 279–292. doi: 10.2307/1369146

[ref68] Roberts J. R. Hughes M. R. (1984). The effects of hypertonic sodium chloride injection on body water distribution in ducks (*Anas platyrhynchos*), gulls (*Larus glaucescens*), and roosters (*Gallus domesticus*). Comp. Biochem. Physiol. 52, 21–28. doi: 10.1016/s0300-9629(75)80120-4 240552

[ref69] Robinson G. A. Martin K. A. Wasnidge D. C. Floto F. (1979). Lipovitellin and phosvitin as major ^140^La-binding components of Japanese quail egg yolk *in vivo* and *in vitro*. Poult. Sci. 58, 1361–1366. doi: 10.3382/ps.0581361

[ref70] Robinson G. A. Wasnidge D. C. Floto F. (1978). Distribution of ^140^La and ^47^Ca in female Japanese quail and in the eggs laid. Poult. Sci. 57, 190–196. doi: 10.3382/ps.0570190, PMID: 674005

[ref71] Robinson G. A. Wasnidge D. C. Floto F. (1980). Radiolanthanides as markers for vitellogenin-derived proteins in the growing oocytes of the Japanese quail. Poult. Sci. 59, 2312–2321. doi: 10.3382/ps.0592312, PMID: 7193323

[ref72] Rząsa J. Hrabia A. Paczoska-Eliasiewicz H. Sechman A. (2008). Changes in blood flow through the chicken ovary and oviduct after serotonin treatment. Bull. Vet. Inst. Pulawy 52, 241–244.

[ref74] Sapirstein I. A. Hartman F. A. (1959). Cardiac output and its distribution in the chicken. Am. J. Phys. 196, 751–752. doi: 10.1152/ajplegacy.1959.196.4.751, PMID: 13637206

[ref75] Scanes C. G. (2022). “Blood,” in Sturkie’s avian physiology. 7th Edn. eds. Scanes C. G. Dridi S. (London: Academic Press), 293–326.

[ref77] Schade R. Chacana P. A. (2007). “Livetin fractions (IgY),” in Bioactive Egg Compounds. eds. Huopalahti R. López-Fandiño R. Anton M. Schade R. (Berlin: Springer-Verlag), 25–32.

[ref78] Seong P. M. Cho S. H. Park K. M. Kang G. H. Park B. Y. Moon S. S.. (2015). Characterization of chicken by-products by mean of proximate and nutritional compositions. Korean J. Food Sci. Anim. Resour. 35, 179–188. doi: 10.5851/kosfa.2015.35.2.179, PMID: 26761826 PMC4682518

[ref79] Sethi P. K. White C. A. Cummings B. S. Hines R. N. Muralidhara S. Bruckner J. V. (2016). Ontogeny of plasma proteins, albumin and binding of diazepam, cyclosporine, and deltamethrin. Pediatr. Res. 79, 409–415. doi: 10.1038/pr.2015.237, PMID: 26571224

[ref80] Shang Y. Regassa A. Ji H. K. Kim W. K. (2015). The effect of dietary fructooligosaccharide supplementation on growth performance, intestinal morphology, and immune responses in broiler chickens challenged with salmonella Enteritidis lipopolysaccharides. Poult. Sci. 94, 2887–2897. doi: 10.3382/ps/pev275, PMID: 26467012

[ref81] Sjöholm I. Ekman B. O. Kober A. Ljungstedt-Påhlman I. Seiving B. Sjödin T. (1979). Binding of drugs to human serum albumin: XI. The specificity of three binding sites as studied with albumin immobilized in microparticles. Mol. Pharmacol. 16, 767–777. PMID: 530258

[ref82] Stifani S. Barber D. L. Nimpf J. Schneider W. J. (1990). A single chicken oocyte plasma membrane protein mediates uptake of very-low-density lipoprotein and vitellogenin. Proc. Natl. Acad. Sci. U. S. A. 87, 1955–1959. doi: 10.1073/pnas.87.5.1955, PMID: 2308956 PMC53603

[ref84] Sturkie P. D. Abati A. (1975). Blood flow in the meseteric, hepatic portal and renal portal veins of chickens. Pfügers Arch. 359, 127–135. doi: 10.1007/BF00581282, PMID: 1239725

[ref85] Sturkie P. D. Newman H. J. (1951). Plasma proteins of chickens as influenced by time of laying, ovulation, number of blood samples taken and plasma volume. Poult. Sci. 30, 240–248. doi: 10.3382/ps.0300240

[ref86] Trampel D. W. Sell J. L. Ahn D. U. Sebranek J. G. (2005). Preharvest feed withdrawal affects liver lipid and liver color in broiler chickens. Poult. Sci. 84, 137–142. doi: 10.1093/ps/84.1.137, PMID: 15685953

[ref87] U.S. Environmental Protection Agency (2004). Overview of the Ecological Risk Assessment Process in the Office of Pesticide Programs: Endangered and Threatened Species Effects Determinations. Available at: https://www.epa.gov/sites/production/files/2014-11/documents/ecorisk-overview.pdf (Accessed December 24, 2020).

[ref88] U.S. Environmental Protection Agency (2019). EPA Releases Draft Policy to Reduce Pesticide Testing on Birds. Available at: https://www.epa.gov/newsreleases/epa-releases-draft-policy-reduce-pesticide-testing-birds (Accessed December 12, 2020).

[ref89] Vogel J. A. Sturkie P. D. (1963). Effects of starvation on the cardiovascular system of the chicken. Proc. Soc. Exp. Biol. Med. 112, 111–113. doi: 10.3181/00379727-112-27965, PMID: 13997638

[ref90] Wang Y.-S. Li M. Tell L. A. Baynes R. E. Davis J. L. Vickroy T. W.. (2020). Physiological parameter values for physiologically based pharmacokinetic models in food-producing animals. Part II: Chicken and turkey. J. Vet. Pharmacol. Therap. 44, 423–455. doi: 10.1111/jvp12931, PMID: 33289178 PMC8359335

[ref91] Watkins E. J. Butler P. J. Kenyon B. P. (2004). Posthatch growth of the digestive system in wild and domesticated ducks. Br. Poult. Sci. 45, 331–341. doi: 10.1080/00071660410001730824, PMID: 15327119

[ref92] Wolfenson D. Frei Y. F. Snapir N. Berman A. (1981). Heat stress effects on capillary blood flow and its redistribution in the laying hen. Pflugers Arch. 390, 86–93. doi: 10.1007/BF00582717, PMID: 7195555

[ref93] Wu Y. N. Yan F. Hu J. Y. Chen H. Tucker C. M. Green-Miller A. R.. (2017). The effect of chronic ammonia exposure on acute-phase proteins, immunoglobulin, and cytokines in laying hens. Poult. Sci. 96, 1524–1530. doi: 10.3382/ps/pew454, PMID: 28379573

[ref94] Yang F. Zhang Y. Liang H. (2014). Interactive association of drugs binding to human serum albumin. Int. J. Mol. Sci. 15, 3580–3595. doi: 10.3390/ijms15033580, PMID: 24583848 PMC3975355

[ref95] Yu J. Y.-L. Marquardt R. R. (1973). Effects of estradiol and testosterone on the immature female chicken (*Gallus domesticus*)—I. Quantitative changes in nucleic acids, proteins and lipids in the liver. Comp. Biochem. Physiol. B 46, 749–757. doi: 10.1016/0305-0491(73)90119-3, PMID: 4128504

[ref96] Yuan C. Bu X. C. Yan H. X. Lu J. J. Zou X. T. (2016). Dietary L-arginine levels affect the liver protein turnover and alter the expression of genes related to protein synthesis and proteolysis of laying hens. Poult. Sci. 95, 261–267. doi: 10.3382/ps/pev314, PMID: 26628340

[ref97] Zaefarian F. Abdollahi M. R. Cowieson A. Ravindran V. (2019). Avian liver: the forgotten organ. Animals 9:63. doi: 10.3390/ani9020063, PMID: 30781411 PMC6406855

