# Supplementary material for: Quantitative Morphometric, Physiological, and Metabolic Characteristics of Chickens and Mallards for Physiologically Based Kinetic Model Development
Source: Front Physiol. 2022 Apr 6;13:858283. doi: 10.3389/fphys.2022.858283 (PMC9019682; doi:10.3389/fphys.2022.858283)
Supplement: Supplementary file 1 [file Data_Sheet_1.docx]

**SUPPLEMENTARY DATA**

**SUPPLEMENTARY TABLE 1.** Cardiac Output in chickens

| Type/age | Cardiac Output  mL min^-1^ kg^-1^ | Cardiac Output  ml min^-1^ | Reference |
| --- | --- | --- | --- |
| **Broiler chickens**^A^ | |  |  |
|  | 174 | 492 | Wideman et al., 1996 |
| 4 weeks old | 208 | 253 | Wideman,1999 |
| 5 weeks old | 193 | 348 | Wideman, 1999 |
| 6 weeks old | 188 | 434 | Wideman, 1999 |
|  | 198 | 471 | Wideman et al., 2000 |
|  |  |  |  |
| **Laying hens** | |  |  |
|  | 218 | 499 | Sapirstein and Hartman, 1959 |
|  | 227 | 363 | Boelkins et al., 1973. |
|  | 137 | 277 | Moynihan and Edwards, 1975 |
|  | 160^B^ | 257 | Niezgoda et al., 1982 |
|  | 113 | 208 | Niezgoda et al., 1979 |
|  | 179 | 287 | Hrabia et al., 2005 |
|  | 201 | 321 | Rząsa et al., 2008 |
|  |  |  |  |
| **Adult male chicken** |  |  |  |
|  | 186 | 411 | Vogel and Sturkie, 1963 |
|  | 150 | 349 | Merrill et al., 1981 |
|  |  |  |  |
| **Ducks** |  |  |  |
| Domestic duck (*Anas platyrhynchos*) | |  |  |
|  | 529 | 1509 | Folkow et al., 1967 |
|  | 304 | 897 | Grubb, 1982 |
|  |  |  |  |
| Tufted duck (*Aythya fuligula*) | |  |  |
|  | 342 | 208 | Bevan and Butler, 1992 |

^A^ Anesthetized

^B^ Assumes body weight of laying chicken is 1.6 kg

**SUPPLEMENTARY TABLE 2.** Studies reporting hematocrit and blood hemoglobin concentrations in sexually immature and mature female chickens together with adult male chickens

|  | Pullet [males] | Laying hens | Reference |
| --- | --- | --- | --- |
| **Hematocrit** |  |  |  |
|  | 31.1 | 29.6 | Medway and Kare, 1959 |
|  |  | 28.6 | Koji, 1960 |
|  |  | 31.5 | Abou-Ashour and Edwards, 1972 |
|  | 29 [45] | 29 | Sturkie, 1986 citing Newell and Schaffner |
|  | [40.8] | 25.5 | Hunsaker, 1969 |
|  | 25.0 | 23.7 | Schaal et al., 2016 |
|  |  | 29.0 | Pantaya and Utami, 2018 |
|  |  | 25.6 | López et al., 2018 |
|  |  | 23.3 | Sauer et al., 2019 |
|  | 27.6 [33.9] | 26.3 | Arnaudov et al., 2020 |
| Mean + (n = studies) SEM | 28.1 + (4) 1.78  [39.9 + (3) 3.24] | 27.1 + (10) 0.86 |  |
| **Hemoglobin** |  |  |  |
|  |  | 9.28 | Abou-Ashour and Edwards, 1972 |
|  | 8.5 | 8.1 | Schaal et al., 2016 |
|  |  | 11.4 | López et al., 2018 |
|  |  | 7.96 | Sauer et al., 2019 |
|  | 9.27 [10.76] | 9.16 | Arnaudov et al., 2020 |
| Mean + (n = studies) SEM | 8.88 + (2) 0.38 | 9.18 + (5) 0.62 |  |

**SUPPLEMENTARY TABLE 3.** Studies reporting hematocrit and blood hemoglobin concentrations in young (4-6 weeks old) broiler chickens

| **Broiler chickens (male and female)** | Parameter | Reference |
| --- | --- | --- |
| **Hematocrit %** |  |  |
|  | 29.1 | Kubena et al., 1972 |
|  | 36.2 | Goodwin et al., 1992 |
|  | 28.24 | Yersin et al., 1992 |
|  | 30 | Furlan et al., 1993 |
|  | 34.1 | Altan et al., 2000 |
|  | 30.73 | Orawan and Aengwanich, 2007 |
|  | 34.0 | Tehrani et al., 2012 |
|  | 29.9 | Onyishi et al., 2017 |
|  | 30.2 | de Carvalho et al., 2020 |
| **Mean + (n=studies) SEM** | **31.4 + (9) 0.90** |  |
| **Hemoglobin** |  |  |
|  | 9.03 | Kubena et al., 1972 |
|  | 9.08 | Yersin et al., 1992 |
|  | 11.6 | Furlan et al., 1993 |
|  | 8.22 | Orawan and Aengwanich, 2007 |
|  | 10.6 | Tehrani et al., 2012 |
|  | 7.43 | de Carvalho et al., 2020 |
| **Mean + (n=studies) SEM** | **9.33 + (6) 0.63** |  |

**SUPPLEMENTARY TABLE 4.** Studies reporting hematocrit and blood hemoglobin concentrations in mallard and domesticated duck

|  | Hct | Hemoglobin | Reference |
| --- | --- | --- | --- |
| Mallard |  |  |  |
|  | 45.7 | 13.9 | Shave and Howard, 1971 |
|  | 40.2 | 15.2 | Driver, 1982 |
|  | 47.5 | 17.1 | Keijer and Butler, 1982 |
|  | 48.3 |  | Cited Zapletal et al., 2017 |
| Mean + (N = studies) SEM | 45.4 + (4) 1.82 | 15.4 + (3) 0.93 |  |
|  |  |  |  |
| Domestic duck |  |  |  |
|  | 39.8 | 14.2 | Mean of Hemm and Carlton, 1967 |
|  | 44.2 |  | Sturkie, 1986 |
|  | 46.7 |  | Sturkie, 1986 |
|  | 40.7 |  | Sturkie, 1986 |
|  | 38.1 |  | Sturkie, 1986 |
|  | 46 |  | Sturkie, 1986 |
|  | 35.5 |  | Zapletal et al., 2017 |
|  | 40.7 | 13.3 | Bhattacherjee et al., 2018 |
|  | 37.2 | 12.7 | Bhattacherjee et al., 2018 |
|  |  | 14.2 | Sturkie, 1986 |
|  |  | 12.7 | Sturkie, 1986 |
|  |  | 12.7 | Sturkie, 1986 |
| Mean + (N = studies) SEM | 41.0 + (9) 1.30 | 13.3 + (6) 0.30 |  |

**SUPPLEMENTARY TABLE 5**. Studies reporting plasma/serum concentrations of albumen and immunoglobulins in chickens

|  | Concentration | Reference |
| --- | --- | --- |
| **ALBUMEN g dL^-1^** |  |  |
| Young chickens/pullets | 1.72 | Morgan, 1975 |
| Young female chicken | 1.70 | Peltonen and Sankari, 2011 |
| Broilers (< 7 weeks old) | 1.42 | Bowles et al., 1989 |
| Broilers (< 7 weeks old) | 1.95 | Meluzzi et al., 1992 |
| Broilers (< 7 weeks old) | 1.56 | Liu et al., 2020 |
|  |  |  |
| Laying hens | 1.10 | Patterson et al., 1962 |
| Laying hens | 1.46 | Morgan, 1975 |
| Laying hens | 1.74 | Peltonen and Sankari, 2011 |
| Laying hens | 0.781 | Yuan et al., 2016 |
| Laying hens | 0.880 | Chen et al., 2017 |
|  |  |  |
| **IgY g dL^-1^** |  |  |
| **Young chickens/pullets** | 0.0948 | Murai et al., 2020 |
|  |  |  |
| **Studies employed in analysis** | | |
| Laying hens | 0.45 | Patterson et al., 1962 |
| Laying hens | 0.464 | Hamal et al., 2006 |
| Laying hens | 0.704 | Strong, 2014 |
| Laying hens | 0.555 | Agrawal et al., 2016 |
| Laying hens | 0.555 | Chen et al., 2017 |
| Laying hens | 0.4052 | Risk et al., 2018 |
| **Studies not employed in analysis due extremely high and low values** | | |
| Laying hens | 0.191 | Carlander et al., 2001 |
| Laying hens | 0.0797 | Çetin et al., 2010 |
| Laying hens | 2.104 | Geng et al., 2018 |
|  |  |  |
| **IgA g dL^-1^** |  |  |
| Young chickens/pullets | 0.0342 | Murai et al., 2020 |
|  |  |  |
| Laying hens | 0.0323 | Hamal et al., 2006 |
| Laying hens | 0.0237 | Chen et al., 2017 |
|  |  |  |
| **IgM g dL^-1^** |  |  |
| Laying hens | 0.0917 | Hamal et al., 2006 |
| Laying hens | 0.172 | Çetin et al., 2010 |
| Laying hens | 0.0374 | Chen et al., 2017 |
| Laying hens | 0.0253 | Geng et al., 2018 |

**SUPPLEMENTARY TABLE 6.** Reproductive and related organ weights in reproductively mature and immature female chickens

| Fat pad | Ovary | Oviduct | Liver | Reference |
| --- | --- | --- | --- | --- |
| **Pullet (immature female chickens)** | | | | |
|  | 0.03 |  |  | Maurice et al., 1982 |
| 0.7 | 0.03 |  |  | Sun et al., 2006 |
|  | 0.09 | 0.07 |  | Martinez et al., 2015 |
|  | 0.033 | 0.022 |  | Dunn et al., 2017 |
|  |  |  | 2.0 | Frikha et al., 2009 |
|  |  |  | 2.8 | Saldaña et al., 2015 |
|  |  |  | 2.9 | Guzmán et al., 2015 |
|  |  |  | 1.79 | Martinez et al., 2013 |
|  |  |  | 1.5 | Martinez et al., 2015 |
|  |  |  | 1.5 | Dou et al., 2017 |
|  |  |  | 0.9 | Dou et al., 2017 |
|  |  |  | 1.6 | Lorenz et al., 1938 |
|  |  |  | 1.69 | An et al., 1997 |
|  |  |  | 2.73 | Trampel et al., 2005 |
|  |  |  | 2.96 | Musundire et al., 2018 |
|  |  |  | 2.09 | Neill et al., 1977 |
|  |  |  |  |  |
| **Laying hen (reproductively mature female chickens)** | | | | |
|  |  |  | 3.17 | Boelkins et al., 1973 |
|  | 1.25 |  | 2.0 | Brody et al., 1984 |
|  | 2.04 | 2.94 | 1.94 | Chen et al., 2007 |
|  |  |  | 3.26 | El-Din et al., 2018 |
|  | 3.8 |  | 3.15 | Emiola et al., 2011 |
|  |  |  | 1.80 | Esonu et al., 2006 |
|  |  |  | 2.03 | Gao et al., 2014 |
|  |  |  | 2.83 | Gregory and Robins, 1998 |
|  | 2.87 | 2.88 | 2.68 | Hassan et al., 2016 |
|  |  |  | 1.91 | Hopkins and Biely, 1935 |
|  | 2.26 | 1.82 | 1.91 | Joseph et al., 2000 |
|  |  |  | 2.32 | Khan et al., 2010 |
|  | 3.95 | 3.84 |  | Kwakkel et al., 1995 |
|  |  |  | 2.56 | Lorenz et al., 1938 |
|  | 1.95 |  | 2.35 | Machebe et al., 2013 |
|  |  |  | 1.96 | Musundire et al., 2018 |
|  | 2.19 | 2.84 | 2.10 | Nassar et al., 2017 |
|  | 2.32 | 3.10 | 1.99 | Nassar et al., 2017 |
|  |  |  | 2.88 | Neill et al., 1977 |
|  | 2.95^A^ | 2.49^A^ |  | Niezgoda et al., 1982 |
|  |  |  | 1.61 | Ogungbesan et al., 2014 |
| 1.83 | 1.82 | 2.12 | 1.62 | Pishnamazi et al., 2014 |
|  | 2.44 |  |  | Saki et al., 2014^a^ |
|  |  |  | 2.65 | Salmi et al., 2006 |
| 4.4 | 1.53 |  |  | Sun et al., 2006 |
|  | 2.12 |  |  | Sun et al., 2015 |
|  |  |  | 2.33 | Wolford and Polin, 1972 |
| 5.09 | 2.83 | 3.21 | 2.48 | Youssef et al., 2016 |

^A^ Assumes body weight of laying chicken is 1.6 kg

**SUPPLEMENTARY TABLE 7.** Organ weights in reproductively mature and immature female chickens

| Organ weights as % of b.wt. | | | | | Reference |
| --- | --- | --- | --- | --- | --- |
| Heart | Gizzard | Kidney | Spleen | Small intestine |  |
| **Pullet (sexually immature female chickens)** | | | | | |
|  | 3.46 |  |  |  | Frikha et al., 2009 |
|  | 1.68 |  | 0.125 |  | Maurice et al., 1982 |
|  | 1.2 |  | 0.12 |  | Kokoszyński et al., 2017 |
|  | 3.94 |  |  |  | Saldaña et al., 2015 |
|  | 4.3 |  |  |  | Guzmán et al., 2015 |
|  | 5.24 |  | 0.26 |  | Guzmán et al., 2015 |
|  | 3.33^P^ | 0.65 | 0.19 | 5.79 | Martinez et al., 2013 |
|  | 1.4 | 0.6 | 0.25 | 2.6 | Martinez et al., 2015 |
|  | 1.4 | 0.6 | 0.25 | 1.795 | Dou et al., 2017 |
|  | 0.83 | 0.26 | 0.12 |  | Dou et al., 2017 |
|  | 6.86 | 1.15 | 0.095 |  | Musundire et al., 2018 |
| **Laying hen** | | | | | |
|  |  | 1.002 | 0.13 |  | Boelkins et al., 1973 |
| 0.43 |  | 0.1 |  |  | Machebe et al., 2013 |
| 0.75 |  | 1.49 | 0.68 |  | El-Din et al., 2018 |
| 0.40 | 0.31 |  |  |  | Esonu et al., 2006 |
| 0.38 | 0.31 | 0.58 | 0.11 |  | Gao et al., 2014 |
| 0.40 | 1.59 |  |  | 3.0 | Salmi et al., 2006 |
| 0.42 | 3.43 | 0.58 | 0.08 | 5.85 | Khan et al., 2010 |
| 0.37 | 2.63 | 0.06 |  | 3.59 | Ogungbesan et al., 2014 |
|  |  | 0.645 | 0.119 |  | Hopkins and Biely, 1935 |
| 0.38 |  | 0.58 | 0.11 |  | Gao et al 2014 |
| 0.48 | 6.27 | 1.24 | 0.17 |  | Emiola et al., 2011 |
|  | 7.91 | 0.75 | 0.094 |  | Musundire et al., 2018 |

^P^ Assumed as other data in the report were out by a factor of 10

**SUPPLEMENTARY TABLE 7A**. Gastro-intestinal organ weights in broiler chickens (>13 days old)

| Proventriculus  % g 100g^-1^ | Gizzard  % g 100g^-1^ | Small intestine % g 100g^-1^ | Reference^A^ |
| --- | --- | --- | --- |
|  | 4.2 | 4.35 | Iji et al., 2001 |
| 1.12 | 4.98 |  | Zavarize et al., 2012 |
|  |  | 3.98 | Moghaddam and Alizadeh-Ghamsari, 2013 |
|  |  | 2.97 | Wu et al., 2013 |
| 0.36 | 1.3 | 3.57 | Aguila et al., 2014 |
| 0.48 | 1.765 | - | Alshamy et al., 2018 |
| 0.63 | 3.71 | 4.04 | Li et al., 2018 |
| 0.42 | 1.64 | 1.73 | Lan et al., 2020 |
|  |  | 4.49 | Pirgozliev et al., 2020 |
| 0.602 + (5) 0.14 | 2.932 + (6) 0.64 | 3.59 + (7) 0.36 | Mean + (n = ) SEM |

^A^ Reports in which proventriculus, gizzard and small intestine weights or relative weights were either found by the following search parameters: proventriculus or gizzard or small intestine AND broiler chicken AND scholarly or employed the studies listed in supplementary table 9. Data from studies where the birds were <13 days old were omitted as relative weights declined in the first 2 weeks of post-hatching life. Relative weights were either as reported or calculated from the weights and body weights.

**SUPPLEMENTARY TABLE 8.** Comparison of lengths of components (in cm) of the gastro-intestinal tract in chickens

| Parameter | Duodenum | Jejunum | Ileum | Caeca | Colon/  rectum | References |
| --- | --- | --- | --- | --- | --- | --- |
| 21 d broiler chickens | 11.27 | 25.04 | 28.07 |  |  | Wu et al., 2013 |
| 23 d broiler chickens^1^ | 22.8 | 41.0 | 38.9 |  |  | de Verdal et al., 2010 |
| 28 d broiler chickens | 34.1 | 123 | 31 |  |  | Nasrin et al., 2012 |
| 5 wk broiler chickens | 32.0 | 70.6 | 66.5 | 33.9 | 10.1 | Alshamy et al., 2018 |
| 5 wk broiler chickens |  |  |  | 40.5 | 11.3 | Kokoszyński et al., 2017 |
| 6 wk broiler chicken | 28.1 | 85.8 | 20.7 | 13.8 | 6.7 | Metzler-Zebeli et al., 2018 |
| 9 wk chickens^2^ | 30.2 | 58.7 | 51.5 | 29.7 | 9.9 | Alshamy et al., 2018 |
| 12 wk white Leghorn male chickens | 22.0 | 47.8 | 43.7 |  |  | Mitjans et al., 1997 |
| Laying hen | 24.3 | 48.8 | 34.5 |  |  | Hurwitz et al., 1973 |
| Laying hen | 27.6 | 56.7 | 54.7 |  |  | Yang et al., 2013 |
| Laying hen | 33.3 | 46.8 | 57.7 | 26.7 | 9.6 | Cutrignelli et al., 2018 |
| Laying hen | 25.12 | 53.92 | 54.24 |  |  | Ding et al., 2018 |
| **Mean + (n = ) SEM** | 26.6 +  (10)  2.1 | 59.3  + (9)  6.0^3^ | 42.7  + (10)  4.6 | 28.1 + (5) 3.9 | 9.48  + (5) 0.72 |  |

**SUPPLEMENTARY TABLE 9.** Small intestine morphometric parameters in chickens

| Parameter | Data | Species and age | Reference |
| --- | --- | --- | --- |
| **Villus Height μm** |  |  |  |
| **Duodenum** |  |  |  |
|  | 674 | Broiler chickens | Xu et al., 2003 |
|  | 1490 | Broiler chickens | Pelicano et al., 2005 |
|  | 1640 | Broiler chickens | Awad et al., 2008 |
|  | 1548 | Broiler chickens | Williams et al., 2008 |
|  | 1467 | Broiler chickens | Incharoen et al., 2010 |
|  | 1928 | Broiler chickens | Lee et al., 2010 |
|  | 1761 | Broiler chickens | Laudadio et al., 2012 |
|  | 870 | Broiler chickens | Nasrin et al., 2012 |
|  | 824 | Broiler chickens | Moghaddam & Alizadeh-Ghamsari, 2013 |
|  | 2058 | Broiler chickens | Chen et al., 2015 |
|  | 1156 | Broiler chickens | Lei et al., 2015 |
|  | 1973 | Broiler chickens | Yue et al., 2015 |
|  | 2058 | Broiler chickens | Bogucka et al., 2017 |
|  | 718 | Broiler chickens | Prakatur et al., 2019 |
|  | 1172 | Indigenous | Mabelebele et al., 2017 |
|  | 619 | Pullet 16 wk old | Li et al., 2018 |
|  | 1040 | 18 wk old males | Yamauchi et al., 2010 |
|  | 1480 | Laying hen | Praes et al., 2011 |
|  | 1309 | Laying hen | Ding et al., 2018 |
| **Broiler Mean = 1440 + (n = 14) SEM 135.4 μm** | | | |
| **Chickens Mean = 1357 + (19) 109.9 μm** | | | |
| **Laying hens Mean = 1394 + (2) 85.5 μm** | | | |
| **Jejunum** |  |  |  |
|  | 3180 | Broiler chickens | Iji et al., 2001 |
|  | 780 | Broiler chickens | Xu et al., 2003 |
|  | 1001 | Broiler chickens | Pelicano et al., 2005 |
|  | 790 | Broiler chickens | Hu and Guo, 2007 |
|  | 848 | Broiler chickens | Incharoen et al., 2010 |
|  | 821 | Broiler chickens | Lee et al., 2010 |
|  | 1161 | Broiler chickens | Laudadio et al., 2012 |
|  | 652 | Broiler chickens | Nasrin et al., 2012 |
|  | 848 | Broiler chickens | Moghaddam and Alizadeh-Ghamsari, 2013 |
|  | 1549 | Broiler chickens | Chen et al., 2015 |
|  | 692 | Broiler chickens | Lei et al., 2015 |
|  | 1055 | Broiler chickens | Wu et al., 2013 |
|  | 1455 | Broiler chickens | Yue et al., 2015 |
|  | 1549 | Broiler chickens | Bogucka et al., 2017 |
|  | 920 | Broiler chicken | Metzler-Zebeli et al., 2018 |
|  | 718 | Broiler chickens | Prakatur et al., 2019 |
|  | 866 | 9 week-old Lohmann dual purpose chickens | Alshamy et al., 2018 |
|  | 1128 | Indigenous | Mabelebele et al., 2017 |
|  | 385 | Pullet 16 wk old | Li et al., 2018 |
|  | 920 | 18 wk old males | Yamauchi et al., 2010 |
|  | 803 | Laying hen | Praes et al., 2011 |
|  | 946 | Laying hen | Cutrignelli et al., 2018 |
|  | 1045 | Laying hen | Ding et al., 2018 |
|  | 1149 | Laying hen | Moniello et al., 2019 |
| **Broiler Mean = 989 + (15) 78.9 μm excluding Iji et al., 2001 as exceeding SD x 10** | | | |
| **Chickens Mean = 960 + (23) 59.0 μm excluding Iji et al., 2001** | | | |
| **Laying hens Mean = 985 + (4) 73.7 μm** | | | |
| **Ileum** |  |  |  |
|  | 541 | Broiler chickens | Xu et al., 2003 |
|  | 919 | Broiler chickens | Pelicano et al., 2005 |
|  | 947 | Broiler chickens | Chichlowski et al., 2007 |
|  | 614 | Broiler chickens | Awad et al., 2008 |
|  | 420 | Broiler chickens | Williams et al., 2008 |
|  | 760 | Broiler chickens | Lee et al., 2010 |
|  | 355 | Broiler chickens | Laudadio et al., 2012 |
|  | 1161 | Broiler chickens | Nasrin et al., 2012 |
|  | 1022 | Broiler chickens | Chen et al., 2015 |
|  | 415 | Broiler chickens | Lei et al., 2015 |
|  | 789 | Broiler chickens | Wu et al., 2013 |
|  | 734 | Broiler chickens | Yue et al., 2015 |
|  | 1022 | Broiler chickens | Bogucka et al., 2017 |
|  | 1075 | Broiler chickens | Kimiaeitalab et al., 2017 |
|  | 377 | Broiler chickens | Alshamy et al., 2018 |
|  | 1172 | Indigenous | Mabelebele et al., 2017 |
|  | 931 | 3 wk. old pullet | Kimiaeitalab et al., 2017 |
|  | 921 | Pullet 16 wk old | Li et al., 2018 |
|  | 540 | 18 wk old males | Yamauchi et al., 2010 |
|  | 630 | Laying hen | Deng et al., 2012 |
| **Broiler Mean = 743 + (15) 71.4 μm** | | | |
| **Chickens Mean = 721 + (20) 59.9 μm** | | | |
| **Laying hens Mean = 630 (1) μm** | | | |
| **Villus width μm** |  |  |  |
| **Duodenum** |  |  |  |
|  | 431 | Broiler chickens | Incharoen et al., 2010 |
|  | 681 | Broiler chickens | Awad et al., 2008 |
|  | 420 | Broiler chickens | Williams et al., 2008 |
|  | 123.3 | Broiler chickens | Nasrin et al., 2012 |
|  | 101.4 | Broiler chickens | Moghaddam and Alizadeh-Ghamsari, 2013 |
|  | 90.9 | Broiler chickens | Laudadio et al., 2012 |
|  | 172.8 | Broiler chickens | Chen et al., 2015 |
|  | 194.3 | Broiler chickens | Bogucka et al., 2017 |
| **Chicken Mean = 276.8 + (8) 74.9 μm** | | | |
| **Jejunum** |  |  |  |
|  | 132 | Broiler chickens | Chichlowski et al., 2007 |
|  | 236 | Broiler chickens | Incharoen et al., 2010 |
|  | 161.3 | Broiler chickens | Laudadio et al., 2012 |
|  | 108.2 | Broiler chickens | Nasrin et al., 2012 |
|  | 92.8 | Broiler chickens | Moghaddam and Alizadeh-Ghamsari, 2013 |
|  | 65 | Broiler chickens | Pelicano et al., 2005 |
|  | 170.6 | Broiler chickens | Chen et al., 2015 |
|  | 218.8 | Broiler chickens | Bogucka et al., 2017 |
| **Chicken Mean = 148.1 + (8) 21.2 μm** | | | |
| **Ileum** |  |  |  |
|  | 105.4 | Broiler chickens | Chichlowski et al., 2007 |
|  | 504 | Broiler chickens | Williams et al., 2008 |
|  | 85 | Broiler chickens | Incharoen et al., 2010 |
|  | 366.2 | Broiler chickens | Laudadio et al., 2012 |
|  | 137.9 | Broiler chickens | Nasrin et al., 2012 |
|  | 163.8 | Broiler chickens | Chen et al., 2015 |
|  | 151.3 | Broiler chickens | Bogucka et al., 2017 |
| **Chicken Mean = 216.2 + (7) 59.3 μm** | | | |

**SUPPLEMENTARY TABLE 10.** Small intestine morphometric parameters in mallards and domestic ducks

|  | Duodenum | Jejunum | Ileum |  |
| --- | --- | --- | --- | --- |
| Villus height | | | | |
| Mallards | 1000 |  |  | Kenyon et al., 2004 |
|  |  |  |  |  |
| Domesticated ducks | 660 |  |  | Kenyon et al., 2004 |
|  | 887 | 847 | 643 | Jiang et al., 2012 |
|  | 700 | 799# | 898 | Wang et al., 2018 |
|  | 1087 | 939 | 699 | Ran et al., 2020 |
| Mean | 891 + (5) 111.7 | 862 + (3) 41.1 | 747 + (3) 77.4 |  |
| Mucosal thickness | 1003 | 898# | 793 | Wang et al., 2018 |
| Small intestine | 107 cm kg^-1^ | | | Jiang et al., 2012 |
| Small intestine | 70.8 cm kg^-1^ | | | King et al., 2000 |

# mean of duodenum and ileum

**SUPPLEMENTARY TABLE11.** Lengths/height (in μm) of villi in the small intestine in mammals

| Species | Duodenum | Jejunum | Ileum | Reference |
| --- | --- | --- | --- | --- |
| Human *(Homo sapiens*) | 704 |  |  | Hasan and Ferguson, 1981 |
| African green monkey (*Chlorocebus aethiops*) | 605 | 663 | 398 | Paulini et al., 2006 |
| Common shrew (Sorex araneus) | 416 | 442 | 356 | Jaroszewska and Wilczyńska, 2006 |
| Guinea pig (*Cavia porcellus*) |  | 579 |  | Weaver and Carrick, 1989; Mitjans, and Ferrer, 2004 |
| Ground squirrel (*Spermophilus tridecemlineatus*) |  | 529 |  | Carey, 1990 |
| Persian squirrel (*Sciurus anomalus*) | 297 | 208 | 203 | Tootian et al., 2013 |
| House mouse (*Mus musculus*) | 386 | 346 | 156 | Short and Derrickson, 2020 |
| Cattle (*Bos taurus*) | 855 | 862 | 583 | Zitnan et al., 2008; Kvidera et al., 2017 |
| Brown rat (*Rattus norvegicus*) | 504 | 416 | 247 | Gardner and Steele, 1989; Seyyedin and Nazem, 2017 |
| Dog (*Canis lupus*) | 739 | 653 | 572 | Verma et al., 1968; Kuzmuk et al., 2005 |
| Pig (*Sus scrofa*) | 663 | 588 | 447 | Kitt et al., 2002; Chwen et al., 2013 |

**SUPPLEMENTARY TABLE 12.** Absorption area in avian model species and various mammalian species

| **Species** | **Absorptive area in m^2^ [cm^2^ x 10^4^]** | **Absorptive area of small intestine in m^2^ kg^-1^** | **Reference** |
| --- | --- | --- | --- |
| **Birds** |  |  |  |
| Chicken | 7.78 | 4.86 | Calculated from Ferrer et al., 1995; Mitjans et al., 1997 |
| Mallard | 5.67 | 7.80 | Watkins et al., 2004 |
| **Mammals** |  |  |  |
| Human (*Homo sapiens*) | 30 | 0.48 | Helander and Fändriks, 2014 |
| Mouse (*Mus musculus*) | 1.105 | 31.5 | Ferraris et al., 1989; Casteleyn et al., 2010 |
| Bat species 1 | 5.42 | 44.4 | Ferraris et al., 1989 |
| Bat species 2 | 29.4 | 47.1 | Ferraris et al., 1989 |
| Rat (*Rattus norvegicus*) | 1.8 | 4.85 | Ferraris et al., 1989 |
| Rabbit (*Oryctolagus cuniculus*) | 12.1 | 5.48 | Ferraris et al., 1989 |
| Dog (*Canis lupus*) | 39.9 | 3.41 | Ferraris et al., 1989 |
| Desert wood rat (*Neotoma lepida*) | 4.1 | 12.2^d^ | Ferraris et al., 1989 |
| **Reptiles** |  |  |  |
| Desert iguana (*Dipsosaurus dorsalis*) | 0.444 | 0.070 | Ferraris et al., 1989 |

**SUPPLEMENTARY TABLE 13.** Bile production in the chicken and multiple mammalian species

| Species | Bile flow mL h^-1^ kg b.w.^-1^ | Reference |
| --- | --- | --- |
| Chicken^M^ | 4.2 | Hurwitz et al., 1973 |
| Chicken | 0.702 | Sturkie, 1976 cited by Duke, 1986 |
| Chicken^N^ | 0.456 | Lisbona et al., 1981 |
| Chicken^O^ | 0.726 | Pullen and Polin, 1984 |
| Chicken | 0.612 | Dukes et al., 1987 |
| Chicken | 0.246 | Dukes et al., 1988 |
| Mean |  |  |
| **Mammals** | | |
| Dog^P^ | 0.6 | Boyer, 1986; 2013 |
| Human^Q^ | 0.347 | Boyer and Bloomer, 1974; Boyer, 1986; 2013; Hundt et al., 2019 |
| Monkey^R^ | 0.9 | Boyer, 1986; 2013 |
| Mouse^S^ | 3 | Bodewes et al., 2015 |
| Pig^T^ | 1.383 | Sambrook, 1981; Martínez et al., 2018 |
| Rabbit^V^ | 5.4 | Boyer, 1986; 2013 |
| Rat^X^ | 4.23 | Boyer, 1986; 2013; Johnson et al., 2002 |
| Sheep | 0.410 | Harrison, 1962 |
| Syrian hamster (*Mesocricetus auratus*) | 2.04 | Robins and Fasulo, 1973 |

^M^ Assumes body weight of 1.5 kg

^N^ Fasted chicken with a mean weight 3.2 kg

^O^ Body weight assumed as 1.8 kg for 8-week old meat type chicken based on National Chicken Council (<https://www.nationalchickencouncil.org/about-the-industry/statistics/u-s-broiler-performance/>)

^P^ Assumes body weight of 15 kg

^Q^ Assumes body weight of 80 kg

^R^ Assumes body weight of 25 kg

^S^ Assumes body weight of 26 g

^T^ Body weight 27.5 kg

^V^ Assumes body weight of 3 kg

^X^ Assumes body weight of 0.3 kg

**SUPPLEMENTARY TABLE 14.** Weights and blood flow to components of the oviduct together with blood flow to the ovary

| Component | Weight | Blood flow  mL min^-1^ | Blood flow  mL min^-1^ g^-1^ | References |
| --- | --- | --- | --- | --- |
| **Infundibulum** |  |  |  |  |
|  | 2.7 |  | 0.352 | Boelkins et al., 1973 |
|  |  | 0.95 |  | Moynihan and Edwards, 1975 |
|  | 3.0 | 0.73 | 0.243 | Niezgoda et al., 1979 |
|  | 2.2 | 0.98 | 0.445 | Wolfenson et al., 1981 |
|  | 2.3 | 0.989 | 0.430 | Niezgoda et al., 1982 |
|  |  | 0.95 |  | Scanes et al., 1982 |
|  |  | 1.11 |  | Hrabia et al., 2005 |
|  |  | 1.32 |  | Rząsa et al., 2008 |
| **Mean + (n =) SEM** | **2.55 + (4) 1.18** | **0.95 + (7) 0.05** | **0.37 + (4) 0.05** |  |
|  |  |  |  |  |
| **Magnum** |  |  |  |  |
|  | 26.2 | 19.7 | 0.752 | Boelkins et al., 1973 |
|  | 20.8 | 4.57 | 0.220 | Moynihan and Edwards, 1975 |
|  | 23.4 | 2.24 | 0.096 | Niezgoda et al., 1979 |
|  | 29.4 | 21.3 | 0.724 | Wolfenson et al., 1981 |
|  | 18.1 | 7.78 | 0.430 | Niezgoda et al., 1982 |
|  |  | 11.25 |  | Scanes et al., 1982 |
|  |  | 10.5 |  | Hrabia et al., 2005 |
|  |  | 9.1 |  | Rząsa et al., 2008 |
| **Mean + (n =) SEM** | **23.6 + (5) 1.98** | **10.8 + (8) 2.37** | **0.44 + (5) 0.13** |  |
|  |  |  |  |  |
| **Isthmus** |  |  |  |  |
|  | 5.9 | 3.6 | 0.610 | Boelkins et al., 1973 |
|  | 4.4 | 3.43 | 0.780 | Moynihan and Edwards, 1975 |
|  | 4.2 | 1.14 | 0.271 | Niezgoda et al., 1979 |
|  | 7.8 | 3.8 | 0.487 | Wolfenson et al., 1981 |
|  | 3.9 | 1.599 | 0.410 | Niezgoda et al., 1982 |
|  |  | 1.57 |  | Scanes et al., 1982 |
|  |  | 2.60 |  | Hrabia et al., 2005 |
|  |  | 2.46 |  | Rząsa et al., 2008 |
| **Mean + (n =) SEM** | **5.24 + (5) 0.73** | **2.52 + (5) 0.36** | **0.511 + (5) 0.09** |  |
|  |  |  |  |  |
| **Uterus** |  |  |  |  |
|  | 9.85 | 19.7 | 2.00 | Boelkins et al., 1973 |
|  | 9.95 | 14.53 | 1.460 | Moynihan and Edwards, 1975 |
|  | 10.2 | 3.37 | 0.330 | Niezgoda et al., 1979 |
|  | 15.4 | 30.8 | 2.00 | Wolfenson et al., 1981 |
|  | 12.3 | 7.8 | 0.634 | Niezgoda et al., 1982 |
|  |  | 8.55 |  | Scanes et al., 1982 |
|  |  | 7.03 |  | Hrabia et al., 2005 |
|  |  | 7.52 |  | Rząsa et al., 2008 |
| **Mean + (n =) SEM** | **11.54 + (5) 1.06** | **12.4 + (8) 3.18** | **1.28 + (5) 0.35** |  |
|  |  |  |  |  |
| **Vagina** |  |  |  |  |
|  | 4.0 | 1.16 | 0.29 | Moynihan and Edwards, 1975 |
|  | 3.5 | 0.77 | 0.22 | Niezgoda et al., 1979 |
|  | 3.3 | 1.06 | 0.321 | Niezgoda et al., 1982 |
| **Mean + (n =) SEM** | **3.60 + (3) 0.21** | **1.00 + (3) 0.12** | **0.28 + (3) 0.03** |  |
|  |  |  |  |  |
| **Ovary** |  |  |  |  |
|  |  | 6.92 |  | Moynihan and Edwards, 1975 |
|  |  | 5.18 |  | Niezgoda et al., 1979 |
|  |  | 5.79 |  | Wolfenson et al., 1981 |
|  |  | 23 |  | Niezgoda et al., 1982 |
|  |  | 3.06 |  | Scanes et al., 1982 |
| **Mean + (n =) SEM** |  | **8.79 + (5) 3.06** |  |  |

**REFERENCES FOR PAPERS CITED IN SUPPLEMENTARY TABLES**

Abou-Ashour, A.M. and Edwards, H.M. 1972. Hematological changes in laying hens receiving *Sterculia foetida* oil supplements. Poult. Sci. 51, 300-305.

Agrawal, R., Hirpurkar, S.D., Sannat, C. and Gupta, A.K. 2016. Comparative study on immunoglobulin Y transfer from breeding hens to egg yolk and progeny chicks in different breeds of poultry. Vet World 9: 425–431.

Aguilar, C.A.L., Lima, K.R. de S., Manno, M.C., Maia, J.G.S., Fernandes Neto, D.L., Tavares, F.B., Roque, T.J.M.R., Mendonça, R. de C.A. and Carmo, E.S.N. do 2014. Rosewood (*Aniba rosaeodora* Ducke) oil in broiler chicken’s diet. Rev. Bras. Saúde Prod. Anim. Salvador 15, 108-119.

Alshamy, Z., Richardson, K.C., Hünigen, H., Hafez, H.M., Plendl, J. and Al Masri, S. 2018. Comparison of the gastrointestinal tract of a dual-purpose to a broiler chicken line: A qualitative and quantitative macroscopic and microscopic study. PLoS ONE 13: e0204921.

Altan, O., Altan, A., Çabuk, M. and Bayraktar, H. 2000. Effects of heat stress on some blood parameters in broilers. Turk. J. Vet. Anim. Sci. 24, 145-148.

Arnaudov, A., Bochukov, A., Petrov, P., V. Gerzilov, V. and Penkov, D. 2020. Study on some hematological and blood biochemical parameters of newly introduced layer breeds depending on the laying period. Trakia J. Sci. 18, Suppl. 1, 11-18.

Awad, W., Ghareeb, K. and Böhm, J. 2008. Intestinal structure and function of broiler chickens on diets supplemented with a synbiotic containing *Enterococcus faecium* and oligosaccharides. Int. J. Mol. Sci. 9: 2205–2216.

Bailey, T.A., Mensah-Brown, E.P., Samour, J.H., Naldo, J., Lawrence, P., Garner, A. 1997. **Comparative morphology of the alimentary tract and its glandular derivatives of captive bustards.** J. Anat. 191: 387-398.

Bevan, R.M. and Butler, P.J. 1992. Cardiac output and blood flow distribution during swimming and voluntary diving of the tufted duck (Aythya Fuligula). J. Exp. Biol. 162, 199-217.

Bhattacherjee, A., Acharya, C.P., Rana, N., Mallik, B.K. and Mohant, P.K. 2013. Haematological and morphometrical analysis of blood cells of Khaki Campbell duck (*Anas platyrhynchos)* in different age groups with respect to sexual dimorphism. Comp. Clin. Pathol. 27, 1465–1472.

Bodewes, F.A.J.A., Bijvelds, M.J., de Vries, W., Baller, J.F.W., Gouw, A.S.H., de Jonge, H.R. and Verkade, H.R. 2015. Cholic acid induces a Cftr dependent biliary secretion and liver growth response in mice. PLoS ONE 10: e0117599.

Boelkins, J.N., Mueller, W.J. and Hall, K.L. 1973. Cardiac output distribution in the laying hen during shell formation. Comp. Biochem. Physiol. A 46:735-743.

Bogucka, J., Dankowiakowska, A., Elminowska-Wenda, G., Sobolewska, A., Jankowski, J., Szpinda, M. and Bednarczyk, M. 2017. Performance and small intestine morphology and ultrastructure of male broilers injected *in ovo* with bioactive substances. Ann. Anim. Sci. 17, 179–195.

Bowles, V.A., Julian, R.J. and Stirtzinger, T. 1989. Comparison of serum biochemical profiles of male broilers with female broilers and white Leghorn chickens. Can. J. Vet. Res. 53, 7-11

Boyer, J.L. 1986. Mechanisms of bile secretion and hepatic transport. In: Physiology of Membrane Disorders pp. 609–636. Eds. T.E. Andreoli, J.F. Hoffman, D.D. Fanestil, S.G. Schultz, Plenum Publishing Corp; New York, NY:

Boyer, J.L. 2013. Bile formation and secretion. Compr. Physiol. 3: 1035–1078.

Boyer, J.L. and Bloomer, J.R. 1974. Canalicular bile secretion in man: Studies utilizing the biliary clearance of (^14^C) mannitol. J Clin Invest. 54, 773–781.

Brody, T.B., Siegel, P.B. and Cherry, J.A. 1984. Age, body weight and body composition requirements for the onset of sexual maturity of dwarf and normal chickens. British Poultry Science 25, 245-252.

Carey, H.V. 1990. Seasonal changes in mucosal structure and function in ground squirrel intestine. Am. J. Physiol. 259, R385–R392.

Carlander, D., Wilhelmson, M. and Larsson, A. 2001. Limited day to day variation of IgY levels in eggs from individual laying hens. Food Agric. Immunol. 13: 87-92.

Casteleyn, C., A Rekecki, A., Van der Aa, A., Simoens, P. and Van den Broeck, W. 2010. Surface area assessment of the murine intestinal tract as a prerequisite for oral dose translation from mouse to man. Lab. Anim. 44: 176–183.

Çetin, Silici, E., Çetin, N. and Güçlü, B.K. 2010. Effects of diets containing different concentrations of propolis on hematological and immunological variables in laying hens. Poult. Sci. 89, 1703-1708.

Chen, H., Huang, R.L., Zhang, H.X., Di, K.Q., Pan, D., Hou, Y. G. 2007. Effects of photoperiod on ovarian morphology and carcass traits at sexual maturity in pullets. Poult. Sci*.* 86, 917–920.

Chen, J., Tellez, G., Richards, J.D. and Escobar, J. 2015. Identification of potential biomarkers for gut barrier failure in broiler chicken. Front. Vet. Sci. 2, 14.

Chen, H., Yan, F.F., Hu, J.Y., Wu, Y., Tucker, C.M., Green, A.R. and Cheng, H.W. 2017. Immune response of laying hens exposed to 30 ppm ammonia for 25 weeks. Int. J. Poult. Sci. 16, 139-146.

Chichlowski, M., Croom, W.J., Edens, F.W., McBride, B.W. Qiu, R., Chiang, C.C., Daniel, L.R., Havenstein, G.B. and Koci, M.D. 2007.Microarchitecture and spatial relationship between bacteria and ileal, cecal and colonic epithelium in chicks fed a direct-fed microbial, PrimaLac and salinomycin. Poult. Sci., 86: 1121-1132.

Chwen, L.T., Foo, H.L., Thanh, N.T. and Choe, D.W. 2013. Growth performance, plasma fatty acids, villous height and crypt depth of preweaning piglets fed with medium chain triacylglycerol. Asian-Australas J. Anim. Sci. 26, 700–704.

Cutrignelli, M.I., Messina, M., Tully, F., Randazzi, B., Olivotto, I., Gasco, L., Loponte, R. and Bovera, F. 2018. Evaluation of an insect meal of the Black Soldier Fly (*Hermetia illucens*) as soybean substitute: Intestinal morphometry, enzymatic and microbial activity in laying hens. Res. Vet. Sci. 117, 208-215.

de Carvalho, G.B., Martins, P.C., Rezende, P.M., Santos, J.S., de Oliveira, E., de Campos Trentin, T.,Martins, D.B., Stringhini, J.H. and Café, M.B. 2020. Hematology and serum biochemistry of broilers at the initial and growth stages submitted to different levels of digestible sulfur amino acids. Ciência Rural, Santa Maria, 50, e20180881.

Denbow, D.M. 2000. Gastrointestinal anatomy and physiology. In: Sturkie’s Avian Physiology 5^th^ ed. pp. 299-325, Ed. G.C. Whittow, Academic Press, San Diego.

Denbow, D.M. 2015. Gastrointestinal anatomy and physiology. In: Sturkie’s Avian Physiology 6^th^ ed. pp. 337-366, Ed. C.G. Scanes, Academic Press, San Diego.

Deng, W., Dong, X.F., Tong J.M. and Zhang Q., 2012. The probiotic *Bacillus licheniformis* ameliorates heat stress-induced impairment of egg production, gut morphology and intestinal mucosal immunity in laying hens. Poult. Sci. 91: 575-582.

de Verdal, H., Mignon-Grasteau, S., Jeulin, C., Le Bihan-Duval, E., Leconte, M., Mallet, S. Martin, C. and Narcy, A. 2010. Digestive tract measurements and histological adaptation in broiler lines divergently selected for digestive efficiency. Poult. Sci. 89:1955–1961.

Ding, X.M., Li, D.D., Bai, S.P., Wang, J.P., Zeng, Q.F.,Su, Z.W., Xuan, Y. and Zhang, K.Y. 2018. Effect of dietary xylooligosaccharides on intestinal characteristics, gut microbiota, cecal short-chain fatty acids, and plasma immune parameters of laying hens. Poult. Sci. 97, 874-881.

Dou, T., Zhao, S., Rong, H., Gu, D., Li, Q., Huang, Y., Xu, Z., Chu, X., Tao, L., Liu, L., Ge, C., te Pas, M. F.W. and Jia, J. 2017. Biological mechanisms discriminating growth rate and adult body weight phenotypes in two Chinese indigenous chicken breeds. BMC Genomics 18: 469.

Driver, E.A. 1981. Hematological and blood chemical values of mallard, *Anas p. platyrhynchos*, drakes before, during and after remige moult. J. Wildl. Dis. 17, 413-421.

Duke, G.E. 1986. Alimentary canal: secretion and digestion, special digestive functions, and absorption. In: Avian Physiology 4^th^ ed. pp. 289-302, Ed. P.D. Sturkie, Springer-Verlag, New York.

Duke, G.E., Larntz, K. and Hunt, H. 1987. The influence of cholecystokinin, vasoactive intestinal peptide and secretin on pancreatic and biliary secretion in laying hens. Comp. Biochem. Physiol. 866: 97-102.

Duke, G.E., Kimmel, J.R., Chaplin, S.B., Hunt, H. and Pollock, H.G. 1988. Influence of avian pancreatic polypeptide on pancreatic and biliary secretion in laying hens. Poult. Sci. 67: 126-130.

Dunn, I.C., Wilson, P.W., Shi, Y., Burt, D.W., Loudon, A.S.I., Sharp, P.J. 2017. Diurnal and photoperiodic changes in thyrotrophin-stimulating hormone β expression and associated regulation of deiodinase enzymes (DIO2, DIO3) in the female juvenile chicken hypothalamus. J. Neuroendocrinol. 29, e12554.

Działa-Szczepańczyk, E. and Wesołowska, I. 2008. **Morphometric characteristics of esophagus and intestine in tufted ducks (**Aythya fuligula**) wintering on the Baltic coastal areas in north-western Poland**. Electron. J. Polish Agric. Univ. 11, 1-35.

El-Din, M.D., Farag, H., Abdallah, E.A., Azeem, A.M.A. and Ahmed, N.A.H. 2018. Wholesomeness and safety of meat of laying hens fed irradiated aflatoxin B1-contaminated diet. Egypt. J. Rad. Sci. Applic. 31, 63 - 75.

Emiola, I.A., Ojebiyi, O.O. and Akande, T.O. 2011. Performance and organ weights of laying hens fed diets containing graded levels of sun-dried cocoa bean shell (CBS). Int. J. Poult. Sci. 10, 986-989.

Esonu, B.O., Opara, M.N., Okoli, I.C., Obikaonu H.O., Udedibie, C. and Iheshiulor, O.O.M. 2006. Physiological response of laying birds to neem (*Azadirachta Indica*) leaf meal- based diets: body weight organ characteristics and haematology. Online J Health Allied Scs. 2:4.

Fairbrother, A., Craig, M., Walker, K. and O’Loughlin, D, 1990. Changes in mallard (*Anas platyrhynchos*) serum chemistry due to age, sex and reproductive condition. J. Wildl. Dis. 26, 67-77.

Ferrer, R., Planas, J.M. and Moretó, M. 1995. Cell apical surface area in enterocytes from chicken small and large intestine during development. Poult. Sci. 74:1995-2002.

Frikha, M., Safaa, H.M., Serrano, M.P., Arbe, X. and Mateos, G.G. 2009. Influence of the main cereal and feed form of the diet on performance and digestive tract traits of brown-egg laying pullets. Poult. Sci. 88: 994-1002.

Furlan, R.L., Tucci, F.M., Nagaghi, L.O., Secato, E.R., Guerreiro, J.R. and Macari, M. 1993. Effect of age and strain on haematological and gasometric parameters in selected and non-selected broiler chickens. Braz. J. vet. Res. anim. Sci. 30, 141-144.

Gao, C., Ma, Q., Zhao, L., Cheng, S. and Ji, C. 2014. Effect of dietary phytase transgenic corn on physiological characteristics and the fate of recombinant plant DNA in laying hens. Asian Australas. J. Anim. Sci.27, 77-82.

Gardner, M.L.G. and Steele, D.L. 1989. Is there circadian variation in villus height in rat small intestine? Quart. J. Exp. Physiol. 74, 257-265.

Geng, Y., Ma, Q., Wang, Z., and Gu, G. 2018. Dietary vitamin D3 supplementation protects laying hens against lipopolysaccharide-induced immunological stress. Nutr. Metab. (Lond.) 15**,** 58.

Goodwin, M.A., Davis, J.F. and Brown, J. 1992. Packed cell volume reference intervals to aid in the diagnosis of anemia and polycythemia in young broiler chickens. Avian Dis. 36, 440-443.

Guzmán, P., Saldaña, B., Kimiaeitalab, M.V., García, J. and Mateos, G.G. 2015. Inclusion of fiber in diets for brown-egg laying pullets: Effects on growth performance and digestive tract traits from hatching to 17 weeks of age. Poult Sci. 94, 2722-2733.

Hamal, K.R., Burgess, S.C., Pevzner, I.Y. and Erf, G.F. 2006. Maternal antibody transfer from dams to their egg yolks, egg whites, and chicks in meat lines of chickens. Poult. Sci. 85: 1364-1372.

Harrison, F.A. 1962. Bile secretion in the sheep. J. Physiol. 162: 212-224.

Hasan, M. and Ferguson, A. 1981. Measurements of intestinal villi in non-specific and ulcer-associated duodenitis correlation between area of microdissected villus and villus epithelial cell count. J. Clin. Pathol. 34, 1181-1186.

Hassan, M.R., Sultana, S., Choe, H.S., Ryo, K.S. 2016. Effect of monochromatic and combined light colour on performance, blood parameters, ovarian morphology and reproductive hormones in laying hens. Ital. J. Anim.Sci. 13,

Helander H. F. and Fändriks L. 2014. Surface area of the digestive tract - revisited. Scand. J. Gastroenterol*.* 49, 681-689.

Hemm, R. and Carlton, W.W. 1967. Review of duck hematology. Poult. Sci. 46, 956-962.

Hena, S.A., Sonfada, M.L., Bello, A., Umar, A.A. 2012. **Some comparative gross and morphometrical studies on the gastrointestinal tract in pigeon (**columbia livia**) and Japanese quail (**coturnix japonica**).** Sci. J. Vet. Adv. 1, 57-64.

Hopkins, J.W. and Biely, J. 1935. Variation in weight of some internal organs of the domestic fowl (*Gallus domesticus*). Can. J. Res. 12, 651-656.

Hrabia, A., Paczoska-Eliasiewicz, H., Niezgoda, J. and Rząsa J. 2005. Histamine affects blood flow through the reproductive organs of the domestic hen (*Gallus domesticus*). Folia Biol (Krakow) 53, 209-213.

Hu, Z. and Guo, Y. 2007. Effects of dietary sodium butyrate supplementation on the intestinal morphological structure, absorptive function and gut flora in chickens. Anim. Feed Sci. Technol. 132, 240–249.

Hundt, M., Basit, H. and Johnm, S. 2019. Physiology, bile secretion. Statpearls. https://www.ncbi.nlm.nih.gov/books/NBK470209/

Hunsaker, W.G. 1969. Species and sex differences in the percentage of plasma trapped in packed cell volume determinations on avian blood. Poult. Sci. 39, 1126-1130.

Hurwitz, S., Bar, A., Katz, M., Sklan, D. and Budowski, P. 1973. Absorption and secretion of fatty acids and bile acids in the intestine of the laying fowl. J. Nutr. 103, 543-547.

Iji, P.A., Saki, A. and Tivey, D.R. 2001. Body and intestinal growth of broiler chicks on a commercial starter diet. 1. Intestinal weight and mucosal development. Br. Poult. Sci. 42: 505-513.

Incharoen, T., Yamauchi, K., Erikawa, T. and Gotoh, H. 2010. Histology of intestinal villi and epithelial cells in chickens fed low-crude protein or low-crude fat diets. Ital. J. Anim, Sci. 9, e82.

Jaroszewska, M. and Wilczyńska, B. 2006. Dimensions of surface area of alimentary canal of pregnant and lactating female common shrews. J. Mammal. 87, 589–597.

Jiang, J.F., Song, X.M., Huang, X., Zhou, W.D., Wu, J.L., Zhu, Z.G., Zheng, H.C. and Jiang, Y.Q. 2012. Effects of alfalfa meal on growth performance and gastrointestinal tract development of growing ducks. Asian-australas. J. Anim. Sci. 25, 1445-1450.

Johnson, D.R., Haseebu, S.M. and Klaassen, C.D. 2002. Increase in bile flow and biliary excretion of glutathione-derived sulfhydryls in rats by drug-metabolizing enzyme inducers is mediated by antidrug resistance protein 2. Toxicol. Sci. 66, 16-26.

Joseph, N.S., Robinson, F.E., Korver, D.R. and Renema, R.A. 2000. Effect of dietary protein intake during the pullet-to-breeder transition period on early egg weight and production in broiler breeders. Poult. Sci. 79, 1790-1796.

Keijer, E. and Butler, P.J. 1982. Volumes of the respiratory and circulating systems tufted and mallard ducks. J. Exp. Biol. 101, 213 -220.

Kenyon, B.P., Watkins, E.J. and Butler, P.J. 2004. Posthatch growth of the digestive system in wild and domesticated ducks. Br. Poult. Sci. 45, 331-341.

Khan, W.A., Khan, M.Z., Khan, A. and Hussain, I. 2010. Pathological effects of aflatoxin and their amelioration by vitamin E in white Leghorn layers. Pakistan Veterinary Journal 30: 155-162.

King, D.E., Asem, E.K. and Adeola, O. 2000. Ontogenetic Development of Intestinal Digestive Functions in White Pekin Ducks. J. Nutr. 130, 57-62.

Kitt, S.J., Miller, P.S., Lewis, A.J. and Fischer, R.L. 2002. Effects of glutamine on growth performance and small intestine villus height in weanling pigs. Nebraska Swine Report. 29-32.

Koji, S. 1960. Hematocrit of chickens; with special reference to visceral lymphomatosis. Poult. Sci. 39, 1126-1130.

Kokoszyński, D., Bernacki, Z., Saleh, M., Stęczny, K. and Binkowska, M. 2017. Body conformation and internal organs characteristics of different commercial broiler lines. Revista Brasileira de Ciência Avícola 19, 47 -51.

Kubena, L.F., May, J.D., Reece, F.N. and Deaton, J.W. 1972. Hematocrit and hemoglobin of broilers as influenced by environmental temperature and dietary iron level. Poult. Sci. 51, 759-763.

Kuzmuk, K.N., Swanson, K.S., Tappenden, K.A., Schook, L.B. and Fahey, G.C. 2005. Diet and age affect intestinal morphology and large bowel fermentative end-product concentrations in senior and young adult dog. J. Nutr. 135, 1940–1945.

Kvidera, S.K., Dickson, M.J., Abuajamieh, M., Snider, D.B., Sanz Fernandez, M.V., Johnson, J.S., Keating, A.F., Gorden, P.J., Green, H.B., Schoenberg, K.M. and Baumgard, L.H. 2017. Intentionally induced intestinal barrier dysfunction causes inflammation, affects metabolism, and reduces productivity in lactating Holstein cows. J. Dairy Sci. 100, 4113–4127.

Kwakkel, R.P., Van Esch, J.A., Ducro, B.J. and Koops, W.J. 1995. Onset of lay related to multiphasic growth and body composition in White Leghorn pullets provided ad libitum and restricted diets. Poult. Sci. 74: 821-832.

Lan, R.X., Li, S.Q., Zhao, Z. and and An, L.L. 2020. Sodium butyrate as an effective feed additive to improve growth performance and gastrointestinal development in broilers. Vet. Med. Sci. 6, 491–499.

Laudadio, V., Passantino, L., Perillo, A., Lopresti, G., Passantino, A., Khan, R. U. and Tufarelli, V. 2012. Productive performance and histological features of intestinal mucosa of broiler chickens fed different dietary protein levels. Poult. Sci. 91:265–270.

Lee, K.W., Lee, S.H. Lillehoj, H.S., Li, G.X. Jang, S.I., Babu, U.S., Park, M.S., Kim, D.K., Lillehoj, E.P., Neumann, A.P., Rehberger, T.G. and Siragusa, G.R. 2010. Effects of direct-fed microbials on growth performance, gut morphometry and immune characteristics in broiler chickens. Poult. Sci. 89: 203-216.

Lei, X., Piao, X., Ru, Y., Zhang, H., Peron, A. and Zhang, H. 2015. Effect of *Bacillus amyloliquefaciens*-based direct-fed microbial on performance, nutrient utilization, intestinal morphology and cecal microflora in broiler chickens. Asian-Aust. J. Anim. Sci., 28: 239-246.

Li, X., Wu, S., Li, X., Yan, T., Duan, Y., Yang, X., Duan, Y., Sun, Q. and Yang, X. 2018. Simultaneous supplementation of bacillus subtilis and antibiotic growth promoters by stages improved intestinal function of pullets by altering gut microbiota. Front. Microbiol. 9, 2328.

Lisbona, J., Jimenéz, Esteller, A. and Lopez, M.A. 1981. Basal biliary secretion in conscious chickens and role of enterohepatic circulation. Comp. Biochem. Physiol. 69A: 341-344.

Liu, J.B., Yan, H.L., Cao, S.C., Hu,Y.D. and H. F. Zhang, H.F. 2020, Effects of absorbents on growth performance, blood profiles and liver gene expression in broilers fed diets naturally contaminated with aflatoxin. Asian-Australas J. Anim. Sci. 33, 294–304.

López, D.P., Huaynate, R.R. and Richard Valles Tananta, R.V. 2018. A comparative evaluation of the hematological parameters, biochemical profile and chemical composition of eggs of creole and Hy-line Brown laying hens. Livestock Research for Rural Development 30 (1) 2018.

Mabelebele, M., Norris, D.I., Brown, D.I.I., Ginindza, M.M. and Ngambi, J.W. 2017. Breed and sex differences in the gross anatomy, digesta pH and histomorphology of the gastrointestinal tract of *Gallus gallus domesticus*. Rev. Bras. Cienc. Avic. 19, 339-346.

Machebe, N.S., Iweh, P., Onyimonyi, A.E., Ekere, O.S. and Abonyi, F. 2013. Zinc oxide as an effective mineral for induced moulting: Effects on post moult performance of laying hens in the humid Tropics. J. Veterinar. Sci. Technol. S11: 003.

Maurice, D.V., Hughes, B.L., Jones, J.E. and Weber, J.M. 1982. The effect of reverse protein and low protein feeding regimens in the rearing period on pullet growth, subsequent performance, and liver and abdominal fat at end of lay. Poult. Sci. 61, 2421-2429.

Martínez, Y., Carrión, Y., Rodríguez, R., Valdivié, M., Olmo, C., Betancur, C., Liu, G., Al-Dhabi, N.A. and Duraipandiyan, V. 2015. Growth performance, organ weights and some blood parameters of replacement laying pullets fed with increasing levels of wheat bran. Rev. Bras. Cienc. Avic. 17, 347-353.

Martínez, G., Diéguez, S.N., Rodríguez, E., Decundo, J.M., Romanelli, A., Fernández Paggi, M.B., Pérez Gaudio, D.S., Amanto, F.L. and Soraci, A.L. 2018. Effect of *Cynara scolymus* and *Silybum marianum* extracts on bile production in pigs. J. Appl. Anim. Res. 46, 1059-1063.

Medway, W. and Kare, M.R. 1959. Blood and plasma volume, hematocrit, blood specific gravity and serum protein electrophoresis of the chicken. Poult. Sci. 38, 624-631.

Meluzzi, A., Primiceri, G., Giordani, R. and Fabris, G. 1992. Determination of blood constituents reference values in broilers. Poult. Sci. 71, 337-345.

Merrill, G.F., Russo, R.E. and Halper, J.M. 1981. Cardiac output distribution before and after endotoxin challenge in the rooster, Am. J. Physiol. 241, R67-R71.

Metzler-Zebeli, B.U., Magowan, E., Hollmann, M., Ball, M.E.E., Molnàr, A., Witter, K.Ertl, R., Hawken, R.J., Lawlor, P.G., O’Connell, N.E., Aschenbach, J. and Zebeli, Q. 2018. Differences in intestinal size, structure, and function contributing to feed efficiency in broiler chickens reared at geographically distant locations. Poult. Sci. 97: 578-591.

Mitjans, M. and Ferrer, R. 2004. Morphometric study of the guinea pig small intestine during development. Misc. Res. Tech. 63, 206-214.

Mitjans, M., Barniol, G. and Ferrer, R. 1997. Mucosal surface area in chicken small intestine during development. Cell Tissue Res. 290:71-78.

Moghaddam, H.N. and Alizadeh-Ghamsari, A.H. 2013. Improved performance and small intestinal development of broiler chickens by dietary L-glutamine supplementation. J. Appl. Anim. Res. 14: 1-7.

Morgan, E.H. 1975. Plasma iron transport during egg laying and after oestrogen administration

in the domestic fowl (*Gallus domesticus*). Quart. J. Exp. Physiol. 60, 233-224.

Moynihan, J.B. and Edwards, N.A. 1975. Blood flow in the reproductive tract of the domestic hen. Comp. Biochem. Physiol 51A, 745-748.

Murai, A., Hamano, T., Kakiuchi, M., Kobayashi, M. and Horio, F. 2020. Evaluation of a receptor gene responsible for maternal blood IgY transfer into egg yolks using bursectomized IgY-depleted chickens. Poult. Sci. 99:1914-1920.

Musundire, M.T., Halimani, T.E. and Chimonyo, M. 2018. Effect of age and sex on carcass characteristics and internal organ weights of scavenging chickens and helmeted guinea fowls. J. Appl. Anim. Res. 46, 860-867.

Nassar, F.S., El-Komy, E.M. and Abdou, A.M. 2017. Ovarian morphology and egg quality traits of Egyptian selected strain for egg production compared with commercial laying strains. Braz.J. Poult. Sci. 19, 683-688

Niezgoda, J., Bobek, S. and Kacińska, 1979. Blood flow in the reproductive tract of the domestic hen following treatment with a pituitary gonadotropic inhibitor. Acta Physiol. Pol. 30: 393-397.

Niezgoda, J., Pierzchała, K., and Bobeks. 1982. Blood flow through the reproductive organs in the hen during the maturation period and egg-laying cycle. Zbl. Vet. Med. A 29, 207-214

Ogungbesan, A., Fasina, O., Adeleke, G. and Alabi, A. 2014. Egg-qualities, organs- weight and carcass-attributes of laying hens (*Gallus domesticus brizzen*) fed *Gliricidia sepium* leaf meal levels supplemented with Maxigrain®. Journal of Biology, Agriculture and Healthcare 4, 37-45.

Onyishi, G.C., Oguine, C.C., Nwani, S.I., Aguzie, I.O. and Nwani, C.D. 2017. Haematological parameters dynamics of developing *Gallus gallus domesticus*. Anim. Res. Int. 2769-2776.

Orawan, C. and Aengwanich, W. 2007. Blood cell characteristics, hematological values and average daily gained weight of Thai indigenous, Thai indigenous crossbred and broiler chickens. Pak. J. Biol. Sci. 10, 302-309.

Pantaya, D. and Utami, M.M.D. 2018. The blood haematological profile on laying hens that treated by different levels of yeast supplementation. IOP Conf. Ser.: Earth Environ. Sci. 207, 012033.

Patterson, R., Youngner, J.S., Weigle, W.O. and Dixon, F.J. 1962. The metabolism of serum proteins in the hen and chick and secretion of serum proteins by the ovary of the hen. J. Gen. Physiol. 45: 501-513.

Paulini, I., Mehta, T. and A Hargis, A. 2006. Intestinal structural changes in African green monkeys after long term psyllium or cellulose feeding. J. Nutr. 117, 253-266.

Pelicano, E.R.L., Souza, P.A., Souza, H.B.A., Figueiredo, D.F., Boiago, M.M., Carvalho, S.R. and Bordon, V.F. 2005. Intestinal mucosa development in broiler chickens fed natural growth promoters. Rev. Bras. Cienc. Avic.7, 221- 229.

Peltonen, L.M. and Sankari, S. 2011. Ott's protein osmotic pressure of serum and interstitial fluid in chickens (*Gallus gallus*): effect of age and gender. J. Exp. Biol. 214: 599-606.

Pirgozliev, V., Westbrook, C., Woods, S., Mansbridge, S.C., Rose, S.P., Whiting, I.M. , Yovchev, D., Atanasov, A.G., Kljak, K., Staykova, G.P., Ivanova, S., Karagecili, M.R., Karadas, F. and Stringhini, J.H. 2020. Feeding dihydroquercetin and vitamin E to broiler chickens reared at standard and high ambient temperatures. Arch. Anim. Nutr. doi: [10.1080/1745039x.2020.1820807](http://dx.doi.org/10.1080/1745039x.2020.1820807)

Pishnamazi, A., Renema, R.A., Zuidhof, M.J., Robinson, F. 2014. Effect of age at photostimulation on sexual maturation in broiler breeder pullets. Poult. Sci. 93, 1274-1281.

Prakatur, I, Miskulin, M., Pavic, M., Marjanovic, K., Blazicevic, V., Miskulin, I. and Domacinovic, M. 2019. Intestinal morphology in broiler chickens supplemented with propolis and bee pollen. Animals (Basel). 9: 301.

Praes, M.F.F.M., Pereira, A.A., Sgavioli, S., Duarte, K.F., Alva, J.C.R., Domingues, C.H. de F., Puzzoti, M.M. and Junqueira, O.M. 2011. Small intestine development of laying hens fed different fiber sources diets and crude protein levels. Rev. Bras. Cienc. Avic. 13, 183-188.

Pullen, D.L. and Polin, D.1984. Effect of bile acids and diet composition on lipid absorption in chickens with cannulated bile ducks. Poult. Sci. 63: 2020-2026.

Ran, M., Hu, B., Cheng, L., Hu, S., Liu, H., Li, L., Hu, J. and Wang, J. 2020. Paternal weight of ducks may have an influence on offspring’ small intestinal function and cecal microorganisms. BMC Microbiol. 20, 145.

Rezende, M.S., Mundim, A.V., Fonseca, B.B., Miranda, R.L., Oliveira W. and Lellis, C.G. 2017. Profile of serum metabolites and proteins of broiler breeders in rearing age. Braz. J. Poult. Sci. 19, 583-586.

Rizk, A.M., El-Sayed, O.A., Qurtam, A.A., Alkilani, W.H. and Ahmed, M.S. 2018. Immunogiobulin IgY transfer from dams to their egg yolks and chicks in some local developed Egyptian strains. Egypt. Poult. Sci. 38: 1141-1154.

Robins, S.J. and Fasulo, J. 1973. Mechanism of lithogenic bile production: studies in the hamster fed an essential fatty acid-deficient diet. Gastroenterology 65, 104-114.

Rząsa, J., Hrabia, A., Paczoska-Eliasiewicz, H and Sechman, A. 2008. Changes in blood flow through the chicken ovary and oviduct after serotonin treatment. Bull. Vet. Inst. Pulawy. 52, 241-244.

Saki, A.A., Aliarabi, H., Siyar, S.A. H., Salari, J. and Hashemi, M. 2014. Effect of a phytogenic feed additive on performance, ovarian morphology, serum lipid parameters and egg sensory quality in laying hen. Vet. Res. Forum. 5, 287–293.

Saldaña, B., Guzmán, P., Cámara, L., García, J. and Mateos, G.G. 2015. Feed form and energy concentration of the diet affect growth performance and digestive tract traits of brown-egg laying pullets from hatching to 17 weeks of age. Poult Sci. 94, 1879-1893.

Salmi, H.E., Senkoylu, N. Akyurek, H. and Agma, A. 2006. Using rice bran in laying hen diets. Central European Journal of Agriculture 7, 135-140.

Sambrook, I.E. 1981. Studies on the flow and composition of bile in growing pigs. J. Sci. Food Ag. 32:781-791.

Sapirstein, I.A. and Hartman, F.A. 1959. Cardiac output and its distribution in the chicken. Am. J. Physiol. 196, 751-752.

Sauer, Z.C.,Taylor, K., Wolc, A., Viall, A.,O’Sullivan, N., Fulton, J.E., Rubinoff, I., Schaal, T. and Sato, Y. 2019. Establishment of Hy-Line commercial laying hen whole blood gas and biochemistry reference intervals utilizing portable i-STAT1 clinical analyzer. Poult. Sci. 98, 2354-2359.

Scanes, C.G., Mozelic, H., Kavanagh, E., Merrill, G. and Rabii, J. 1982. Distribution of blood flow in the ovary of domestic fowl (*Gallus domesticus*) and changes after prostaglandin F-2 alpha treatment. J. Reprod. Fertil. 64: 227-231.

Schaal, T.P., Arango, J., Wolc, A., Brady, J.V., Fulton, J.E., Rubinoff, I., Ehr, I.J., Persia, M.E. and O’Sullivan, N.P. 2016. Commercial Hy-Line W-36 pullet and laying hen venous blood gas and chemistry profiles utilizing the portable i-STAT®1 analyzer. Poult. Sci. 95, 466–471.

Seyyedin, S. and Nazem, M.N. 2017. Histomorphometric study of the effect of methionine on small intestine parameters in rat: an applied histologic study. Folio Morphol. 76, 620-629.

Shave, H.J. and Howard, V., 1976. A hematologic survey of captive waterfowl. J. Wildl. Dis. 12, 195–201.

Short, K. and Derrickson, E.M. 2020. Compensatory changes in villus morphology of lactating *Mus musculus* in response to insufficient dietary protein. J. Exp. Biol. 223, jeb210823.

Stevenson, J. 1933. Experiments on the digestion of food by birds. Wilson Bulletin 45, 155-166.

Sturkie, P.D. 1976. Alimentary canal: anatomy, prehension, deglutition, feeding, drinking, passage of ingesta, and motility. In: Avian Physiology 3^rd^ Ed. pp. 185-195, Ed. P.D. Sturkie, Springer-Verlag, New York.

Sturkie, P.D. 1986. Body fluids: blood. In: Avian Physiology Ed: P.D. Sturkie, pp.102-129. Springer, New York.

Sturkie, P.D. and Newman, H.J. 1951. Plasma proteins of chickens as influenced by time of laying, ovulation, number of blood samples taken and plasma volume. Poult. Sci. 30, 240-248.

Sun, J. M., Richards, M.P. Rosebrough, R.W., Ashwell, C. M., McMurtry, J.P. and Coon, C.N. 2006. The relationship of body composition, feed intake, and metabolic hormones for broiler breeder females. Poult. Sci. 85:1173–1184.

Sun, C., Lu, J., Yi, G. Yuan, J., Duan, Z., Qu, L., Xu, G., Wang, K., Yang, N. 2015. Promising loci and genes for yolk and ovary weight in chickens revealed by a genome-wide association study. PLoS ONE 10, e0137145.

Tehrani, A., Javanbakht, J., Askari, S., Hassan, M.A., Solati, A., Golami, S. and Akbari, H. 2012. Haematological studies on broiler chickens fed with different levels of *Artemia Urmiana*. J Biotechnol Biomater, 2, 4.

Tootian, Z., Sadeghinezhad, J., Sheibani, M.T., Fazelipour, S., De Sordi, N. and Roberto Chiocchetti, R. 2013. Histological and mucin histochemical study of the small intestine of the Persian squirrel (*Sciurus anomalus*). Anat. Sci. Int. 88, 38–45.

Verma, S., Sehgal, A.K., Chakravarti, R.N. and Chhuttan, P.N. 1968. Intestinal villi in the dog and effect of *Ankostoma caninum* infestation. J. Pathol. Bacteriol. 95, 568-571.

Vogel, J. A. and Sturkie, P. D. 1963. Effects of starvation on the cardiovascular system of the chicken. Proc. Soc. Exp. Biol. Med. 112:111-113.

Wang, S., Chen, L., He, M., Shen, J., Li, G., Tao, Z., Wu, R. and Lu, L. 2018. Different rearing conditions alter gut microbiota composition and host physiology in Shaoxing ducks. Sci. Rep. 8**,** 7387.

Watkins, E.J., Butler, P.J. and Kenyon, B.P. 2004. Posthatch growth of the digestive system in wild and domesticated ducks. Br. Poult. Sci. 45:331-341.

Weaver, L.T. and Carrick, B.M. 1989. Changes in upper intestinal epithelial morphology and kinetics in the growing guinea pig. Pediatr. Res. 26, 31-33.

Wideman, R. F. 1999. Cardiac output in four-, five-, and six-week-old broilers, and hemodynamic responses to intravenous injections of epinephrine. Poultry Sci. 78, 392-403.

Wideman, R. F., Y. K. Kirby, C. D. Tackett, N. E. Marson, and R. W. McNew, 1996. Cardio-pulmonary function during acute unilateral occlusion of the pulmonary artery in broilers fed diets containing normal or high levels of arginine-HCl. Poultry Sci. 75, 1587–1602.

Wideman, R. F., Fedde, M. R., Tackett, C. D. and Weigle, G. E. 2000. Cardio-pulmonary function in preascitic (hypoxemic) or normal broilers inhaling ambient air or 100% oxygen. Poult. Sci. 79, 415–425.

Williams, J., Mallet, S., Leconte, M., Lessire, M. and I. Gabriel, I. 2008. The effects of fructo-oligosaccharides or whole wheat on the performance and digestive tract of broiler chickens.

Br. Poult. Sci. 49, 329-339.

Wolfenson, D., Frei, Y.F., Snapir, N. and Berman, A. 1981. Heat stress effects on capillary blood flow and its redistribution in the laying hen. Pflügers Arch. 390, 86-93.

Wu, Q. J., Zhou, Y. M., Wu, Y. N. and Wang, T. 2013. Intestinal development and function of broiler chickens on diets supplemented with Clinoptilolite. Asian-Australas J. Anim. Sci. 26: 987–994.

Wu, Y.N., Yan, F., Hu, J.Y., Chen, H., Tucker, C.M., Green-Miller, A.R. and Cheng, H.W. 2017. The effect of chronic ammonia exposure on acute-phase proteins, immunoglobulin, and cytokines in laying hens. Poult. Sci. 96, 1524-1530.

Xu, Z.R., Hu, C.H., Xia, M.S., Zhan, X.A. and Wang, M.Q. 2003. Effects of dietary fructooligsaccharide on digestive enzyme activities, intestinal microflora and morphology of male broilers. Poult. Sci. 82, 1030-1036.

Yamauchi, K-E., Incharoen, T. and Yamauchi, K. 2010. The relationship between intestinal histology and function as shown by compensatory enlargement of remnants villi after midgut resection in chickens. Anat. Rec. 293, 2071-2079.

Yang, H.M., Wang, W., Wang, Z.Y., Wang, J., Cao, Y.J. and Chen, Y.H. 2013. **Comparative study of intestinal length, weight and digestibility on different body weight chickens**. Afr. J. Biotechnol. 12, 5087-5100.

Yersin, A.G., Huff, W.E., Kubena, L.F., Elissalde, M.H., Harvey, R.B., Witzel, D.A. and Giroir, L.E.

1992. Changes in hematological, blood gas, and serum biochemical variables in broilers during exposure to simulated high altitude. Avian Dis. 36, 189-196.

Yuan, C., Bu, X.C., Yan, H.X., Lu, J.J. and Zou, X.T. 2016. Dietary L-arginine levels affect the liver protein turnover and alter the expression of genes related to protein synthesis and proteolysis of laying hens. Poult. Sci. 95, 261-267.

Yue, S., Regassa, A., Ji, H.K. and Kim, W.K. 2015.The effect of dietary fructooligosaccharide supplementation on growth performance, intestinal morphology, and immune responses in broiler chickens challenged with Salmonella Enteritidis lipopolysaccharides. Poult. Sci. 94. 2887-2897.

Youssef, A.A., El-Hamid, A.E.-H.E., Abedalla, A.A., Berika, M.A., Al-Harthi, M.A., Kucuk, O., Sahin, K. and Abou-Shehema, B.M. 2016. Laying performance, digestibility and plasma hormones in laying hens exposed to chronic heat stress as affected by betaine, vitamin C, and/or vitamin E supplementation. Springer Plus 5, 1619.

Zapletal, D., Kudělková, L., Šimek, V., Petra Jakešová, P., Macháček, M., Eva Straková, E., Suchý, P. 2017. Haematological indicators in hybrid mallard ducks (*Anas platyrhynchos*) with regard to the use of meal from whole white lupin seeds in their diet. Acta Vet. Brno, 86: 309-315.

Zavarize, K.C., Sartori, J.R., Gonzales, E. and Pezzato, A.C. 2012. Morphological changes of the intestinal mucosa of broilers and layers as affected by fasting before sample collection. Braz. J. Poult. Sci. 14, 21-25.

Zitnan, R., Voigt, J., Kuhla, S., Wegner, J., Chudy, A., Schoenhusen, U., Brna, M., Zupcanova, M. and Hagemeister, H. 2008. Morphology of small intestinal mucosa and intestinal weight change with metabolic type of cattle. Veterinarni Medicina 53, 525–532
